# Putative bacterial interactions from metagenomic knowledge with an integrative systems ecology approach

**DOI:** 10.1002/mbo3.315

**Published:** 2015-12-17

**Authors:** Philippe Bordron, Mauricio Latorre, Maria‐Paz Cortés, Mauricio González, Sven Thiele, Anne Siegel, Alejandro Maass, Damien Eveillard

**Affiliations:** ^1^ MathomicsCenter for Mathematical ModelingUniversidad de ChileSantiagoChile; ^2^ Center for Genome Regulation (Fondap 15090007)Universidad de ChileSantiagoChile; ^3^ Laboratorio de Bioinformática y Expresión GénicaINTAUniversidad de ChileSantiagoChile; ^4^ Max Planck Institute for Dynamics of Complex Technical SystemsMagdeburgGermany; ^5^ IRISAUMR 6074CNRSRennesFrance; ^6^ INRIADyliss TeamCentre Rennes‐Bretagne‐AtlantiqueRennesFrance; ^7^ Department of Mathematical EngineeringUniversidad de ChileSantiagoChile; ^8^ LINAUMR CNRS 6241EMNUniversité de NantesNantesFrance

**Keywords:** Environmental microbiology, in silico analysis, metabolic pathways, molecular microbial ecology

## Abstract

Following the trend of studies that investigate microbial ecosystems using different metagenomic techniques, we propose a new integrative systems ecology approach that aims to decipher functional roles within a consortium through the integration of genomic and metabolic knowledge at genome scale. For the sake of application, using public genomes of five bacterial strains involved in copper bioleaching: *Acidiphilium cryptum*,* Acidithiobacillus ferrooxidans*,* Acidithiobacillus thiooxidans*,* Leptospirillum ferriphilum,* and *Sulfobacillus thermosulfidooxidans*, we first reconstructed a global metabolic network. Next, using a parsimony assumption, we deciphered sets of genes, called *Sets from Genome Segments* (SGS), that (1) are close on their respective genomes, (2) take an active part in metabolic pathways and (3) whose associated metabolic reactions are also closely connected within metabolic networks. Overall, this SGS paradigm depicts genomic functional units that emphasize respective roles of bacterial strains to catalyze metabolic pathways and environmental processes. Our analysis suggested that only few functional metabolic genes are horizontally transferred within the consortium and that no single bacterial strain can accomplish by itself the whole copper bioleaching. The use of SGS pinpoints a functional compartmentalization among the investigated species and exhibits putative bacterial interactions necessary for promoting these pathways.

## Introduction

The ecosystems behavior, as observed today by experiments, is the immediate result of microbial interactions between several organisms. By themselves, these interactions explain several steps of natural biogeochemical cycles as well as biological processes of economical interest. Interestingly, recent high‐throughput genomic data have shown for different ecological systems a microbial diversity that was far greater than expected (Roesch et al. 2007; Hingamp et al. 2013; de Vargas et al. 2015) and promise to improve the understanding of microbial ecosystems behaviors from the molecular viewpoint. To this aim, recent studies advocate for the use of metagenomic techniques to intensively investigate different microbial ecosystems (DeLong 2005; Karsenti et al. 2011; Ranjard et al. 2013).

Advances in bioinformatics have also improved the analysis of next‐generation sequencing data that characterize microbial communities, addressing the question “*who is there and who is not*” (Raes et al. 2011). However, this description remains insufficient to depict functional behaviors of microbial ecosystems if no other complementary knowledge is considered. Recent studies overcame this weakness by combining all biotechnological resources available within a modeling framework. In particular, one must notice the success of techniques that target potential cross‐feedings within microbial consortium. Without being exhaustive, they focus on either solely community metabolic network (via graph or constraint based approaches) (Borenstein et al. 2008; Zengler and Palsson 2012; Zomorrodi et al. 2014), or co‐occurrence graph techniques that interconnect covariation of microbial abundance (Faust and Raes 2012; Faust et al. 2012) and environmental features (Ruan et al. 2006, 2010; Brown et al. 2009; Chaffron et al. 2010; Patel et al. 2010). Some other techniques advocate for hybrid approaches that combine heterogeneous knowledge. Microbial cross‐feedings are for instance investigated by integrating phylogenetic and environmental knowledge (see Zaneveld et al. 2011 for review); omics experiments and in situ observations (Orphan 2009; Zelezniak et al. 2015); or metabolic networks and diversity graphs (Tzamali et al. 2011). These recent modeling approaches are all complementary and reinforce the emergence of the new subdiscipline called systems ecology (Klitgord and Segrè 2011) that aims to tackle complex ecological questions by merging heterogeneous data with new computational techniques. However, applications of these techniques remain difficult when communities are experimentally out of reach, which is particularly the case for studying cross‐feedings between extremophile species.

This study overcomes this issue by proposing a modeling framework for metagenomic consortia analysis that not only considers the presence/absence of several bacterial species, but rather their precise genome composition and corresponding metabolic networks. Complementary to previous integrative methods (Segata et al. 2013; Zelezniak et al. 2015), our framework emphasizes putative functional species interactions in the metagenomic consortium through the integration of genome‐wide genomic and metabolic knowledge. For its application, we considered a chemoautotrophic microbial community related to the copper bioleaching process, one of the most extensive and complex biohydrometallurgic processes in which a series of chemical and biological reactions facilitate the oxidation of insoluble sulfide ores, releasing soluble metals such as copper. Beside their economic interest, different theories postulate that these mineral complexes were the principal source of energy used by ancient bacteria consortia (Chemo‐autotrophic Iron‐Sulfur World theory) (Drobner et al. 1990; Wächtershäuser 2000). This particular mining microbial ecosystem is characterized by high concentrations of metals and elevated acidity which advocates for the use of these living microbial species to study adaptation under extreme conditions. Furthermore, the community exhibits a remarkably simple habitat for understanding how ancient microbial communities work (Baker and Banfield 2003). Finally, this microbial ecosystem could be resumed by a limited number of bacterial strains (Yin et al. 2008). This reductionist advantage is of particular interest to benchmark bioinformatics methods on a simple community system while maintaining realistic ecological features.

In terms of current knowledge, several bacteria participating in bioleaching of copper have been isolated and sequenced (Barreto et al. 2003; Mi et al. 2011; Valdés et al. 2011; Travisany et al. 2012). Even though the study of these strains significantly improved the bioleaching knowledge, studying single organisms did not allow to understand bioleaching as a whole. For instance, little is known about the relative functional importance of each bacterium in the bioleaching.

**Figure 1:**
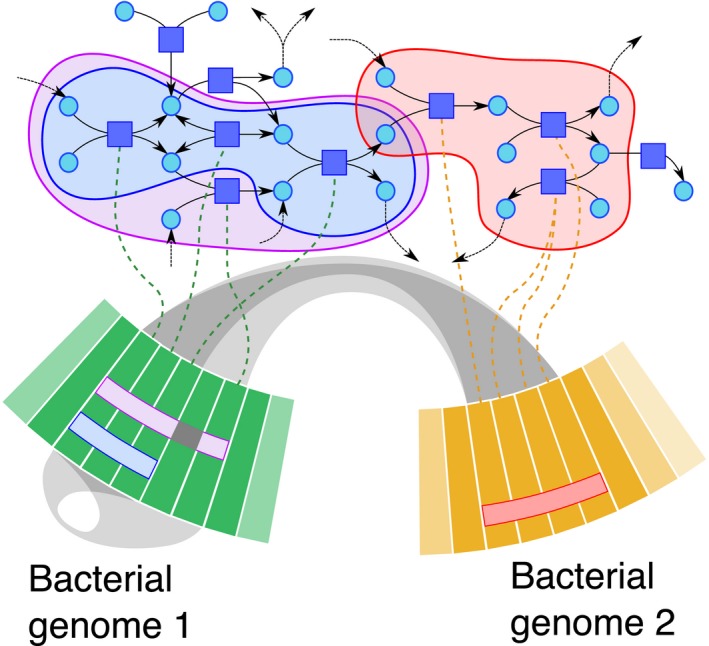
Illustration of *sets from genome segments* (SGS) when applied on a toy microbial community. The upper part of the figure illustrates a metabolic network, where circles are metabolic compounds, and squares are reactions that happen between them. The two bands at the lower part represent parts of two bacterial genomes as sequences of genes. The catalytic function of a gene, via its enzyme, is represented by a dashed line between the gene and the reactions it catalyzes. A SGS appears on the genome as set of genes contained into a segment. The segment containing a SGS can contain genes that do not participate into the SGS (e.g., without catalytic function). The projection of a SGS on the metabolic network defines a set of reactions. Two SGS are linked together by a gray ribbon if they can be chained through the metabolic network.

To decipher the respective genome‐wide role of each bacterium within this mining ecosystem, this work integrates, at community scale, genomic, and metabolic knowledge. Genomic and metabolic features integration relies on a parsimonious assumption that considers the different omics knowledge connected linked by intrinsic and direct properties connections, as adapted from a previous single‐cell systems biology studies (Boyer et al. 2005; Bordron et al. 2011). In practice, our approach connects genomic and metabolic knowledge by considering the genome organization and the biochemical reactions catalyzed by enzymes encoded by its genes. The underline parsimonious principle assumes that genes that must be jointly regulated to activate a metabolic reaction cascade, herein a bioleaching pathway, and should be close enough in the genome organization (for illustration dashed lines in Fig. 1). The corresponding sets of genes satisfying the above mentioned constraints are defined by *Sets from Genome Segments* (SGS) (respectively, pink and blue segments in bacterium 1, and the red segment in bacterium 2 in Fig. 1). SGS represents a segment of consecutive genes in a bacterial genome with a maximum number of genes that participates in a given metabolic pathway. Through this selection, SGS decipher putative sets of genes that (1) take an active part in metabolic pathways while being closely connected via metabolic networks and (2) are consecutive on each of the genomes involved.

We applied this approach to a reduced but exhaustive bacterial community composed of five different copper biomining microbial strains which are simultaneously growing in an industrial bioreactor, fully operational for large‐scale cultures (CODELCO, Radomiro Tomic Division, Antofagasta, Chile): *Acidithiobacillus ferrooxidans ATCC 23270*,* Acidiphilium cryptum JF‐5*,* Acidithiobacillus thiooxidans ATCC 19377*,* Leptospirillum ferriphilum ML‐04,* and *Sulfobacillus thermosulfidooxidans DSM 9293* strains. The selected bacterial strains; beside being the main five mining genomes (sequenced, assembled, and annotated) available today; also cover the four dominant bacterial taxonomic groups present in copper mines, that represents more than the 50% of the total bacterial genera present in mine ecosystems (Yin et al. 2008).

After an exhaustive description of the SGS method and omics data used for its application (e.g., genomes and metabolic networks), this study provides an integrative view of bioleaching at both metabolic pathway and microbial genome levels. In particular, SGS will be enumerated and their relative distribution on all the five genomes of bacteria further detailed. At the metabolic level, SGS modeling paradigm pinpoints evident complementarities of bacterial strains to promote selected bioleaching pathways. In addition, beyond the simple mapping of bacterial strain metabolism on these pathways of interest, SGS depict functional units, similar to operons, that must be combined to genetically monitor the whole bacterial participation in the bioleaching process, as well as specific transporters that should be further investigated to understand putative metabolic collaborative processes at the community level.

## Materials and Methods

### Genomes

Five distinct genomes from bacteria involved in copper bioleaching were considered: *A. cryptum* JF‐5 (NCBI ‐ plasmid & chromosome GenBank ID: from CP000689 to CP000697) (Magnuson et al. 2010), *At. ferrooxidans* ATCC 23270 (NCBI ‐ GenBank ID: CP001219) (Valdes, 2008), *At. thiooxidans* ATCC 19377 (NCBI ‐ GenBank ID: AFOH00000000) (Valdés et al. 2011), *L. ferriphilum* ML‐04 (NCBI ‐ GenBank ID: CP002919) (Mi et al. 2011), and *Sb. thermosulfidooxidans* DSM 9293 (JGI database ‐ Project ID: 97948). The three annotated genomes of *A. cryptum*,* At. Ferrooxidans,* and *L. ferriphilum* are, respectively, composed of 3691 (3158 genes from main chromosome and 533 from plasmids), 3304, and 2527 genes, whereas 2884 and 3602 potential genes were predicted, respectively, for the two nonannotated genomes *At. thiooxidans* and *Sb. thermosulfidooxidans*.

### Metabolic networks

Metabolic networks of *A. cryptum*,* At. Ferrooxidans,* and *L. ferriphilum* were downloaded from Metacyc database v17.5 (Caspi et al. 2012). *Sb. thermosulfidooxidans* and *At. thiooxidans* metabolic networks were reconstruct following a standard procedure: both genome sequences were annotated using a local GenDB platform (Meyer et al. 2003). Gene predictions were made using Glimmer and functional gene annotations were processed by using state‐of‐the‐art methods such as SignalP and TMHMM, and by performing local BLAST searches against databases NCBI nr, Swissprot, Omniome, PDB, KEGG, COG, and TCDB. After gene annotation, a GenBank format file was constructed for each genome and used as a general input for metabolic reconstruction using Pathway Tools software v16.0 (Karp et al. 2010). To reconstruct a metabolic network, we considered a metabolic reaction present when a gene encoding for an enzyme associated to this reaction was identified within the genome. Reactions have then been connected together if a product of a reaction was the substrate of another. To build a metabolic network of the microbial community, the union of the five different metabolic networks was considered.

Complementary, and for the sake of illustration, a list of 13 pathways considered as related to the copper bioleaching process was emphasize (Quatrini et al. 2009): (1) Fe(II) oxidation (Metacyc ID: PWY‐6692); (2) heme biosynthesis: (a) Superpathway of heme biosynthesis from uroporphyrinogen‐III (Metacyc ID: PWY0‐1415), (b) heme biosynthesis from uroporphyrinogen‐III I (Metacyc ID: HEME‐BIOSYNTHESIS‐II), c) heme biosynthesis from uroporphyrinogen‐III II (Metacyc ID: HEMESYN2‐PWY), d) Superpathway of heme biosynthesis from glutamate (Metacyc ID: PWY‐5918), and e) Superpathway of heme biosynthesis from glycine (Metacyc ID: PWY‐5920); (3) iron‐oxidizing/O2‐reducing supercomplex (Metacyc ID: CPLX‐8218); (4) NAD biosynthesis: a) NAD biosynthesis I (from aspartate) (Metacyc ID: PYRIDNUCSYN‐PWY), b) NAD biosynthesis II (from tryptophan) (Metacyc ID: NADSYN‐PWY), and c) NAD biosynthesis III (Metacyc ID: NAD‐BIOSYNTHESIS‐III); (5) Superpathway of sulfate assimilation and cysteine biosynthesis (Metacyc ID: SULFATE‐CYS‐PWY); (6) [2Fe‐2S] iron‐sulfur cluster biosynthesis ([Fe‐S] cluster biosynthesis) (Metacyc ID: PWY‐7250); (7) glutathione biosynthesis (Metacyc ID: GLUTATHIONESYN‐PWY).

#### Sets from Genome Segments modeling framework and associated methods

The metabolism of a given bacterial system is defined by a set of compounds and the related metabolic reactions. A metabolic network was represented as a directed graph, also called metabolic graph, in which nodes represent reactions and an edge between two reactions exists when a product of the input reaction is a substrate of the targeted one. Notice herein that for the sake of modeling and following recommendations in Croes et al. (2005), we have deliberately neglected common and highly connected compounds that are present in the environment (i.e., compounds like water, ATP, NADP, etc., that are present in more than 40 reactions in this paper) and cofactors listed in Christian et al. (2009) to avoid artificial interconnections between reactions (see Ravasz et al. 2002; Guimera and Amaral 2005 for similar assumptions). Complementary, each bacterial chromosome were represented as an ordered list of genes, called *sequence*. Some of these genes encode for enzymes known to catalyze metabolic reactions. We called them metabolic genes. Notice that a given reaction may be catalyzed by several enzymes, implying that a reaction can be associated to one or several metabolic genes.

Assuming a direct causal link between a metabolic gene and a reaction via its encoded enzyme, we designed the SGS paradigm to integrate both knowledge. A SGS is a set of *metabolic genes*, contained in a segment (i.e. a *subsequence*) of the genome, that connects a predetermined initial reaction (and related gene) to a predetermined ending reaction (and related gene) within the metabolic network. Among the whole combination of SGS, our technique seeks to decipher SGS that satisfy the following parsimonious properties (see appendix S1 for a formal definitions and S2 for a parameter sensitivity analysis):

1. For all five bacterial chromosomes, the search was restricted to SGS with at most 20 consecutive metabolic genes (no more SGS were found for more than 20 genes). All pairs of initial and end reactions were considered.
2. From the whole set of SGS, those with a genomic density greater than or equal to 0.6 were selected (no more than ~⅓ of genes are missing): the genomic density is the ratio of the number of genes involved in the metabolic pathway covered by the SGS by the total number of genes within the SGS (see appendix S1 for formal definition). For example, in Figure 1, the purple SGS has a genomic density equal to 0.8, whereas blue and red SGS have a genomic density of 1. When a SGS has a genomic density equal to 1, all genes in the SGS are involved in the targeted metabolic pathway. Conversely, a genomic density close to zero implies that few genes in the SGS are involved in the metabolic pathways.
3. To avoid a “Russian doll effect”, among dense SGS, we selected the *dominant* ones, that are, those that are not included into larger SGS.

### Transcriptomic enrichment

In order to support the SGS prediction, we compared our results to a set of microarray experiments performed on cultures of *At. ferrooxidans* strain Wenelen (DSM 16786 ; Latorre et al. 2015). Wenelen was grown in 62 mmol/L FeSO_4_‐7H_2_O containing modified 9 K medium ((NH_4_)_2_SO_4_: 0.4 g/L; K_2_HPO_4_: 0.4 g/L; MgSO_4_‐7H_2_O: 0.4 g/L) adjusted to pH 1.8 with concentrated sulfuric acid in batch conditions until early exponential phase of the culture (24 h at 30°C). Afterward, four minerals (quartz concentrate (20% p/v), sample of pyrite concentrate (10% p/v), chalcopyrite concentrate (5% p/v) and elemental sulfur powder (5% p/v)) were added to the bacterial cultures (except to the control), which were grown at 30°C without shaking but with forced air supply. After 16 h of grown the cells were collected to RNA extraction, cDNA synthesis and microarrays hybridization. From a transcriptomic viewpoint, the expression for common genes between ATCC23270 and Wenelen strains is identical (Levican et al. 2008; see ortholog list between both strains in File S2).

## Results

### Overlap of metabolic reactions within the community

**Figure 2:**
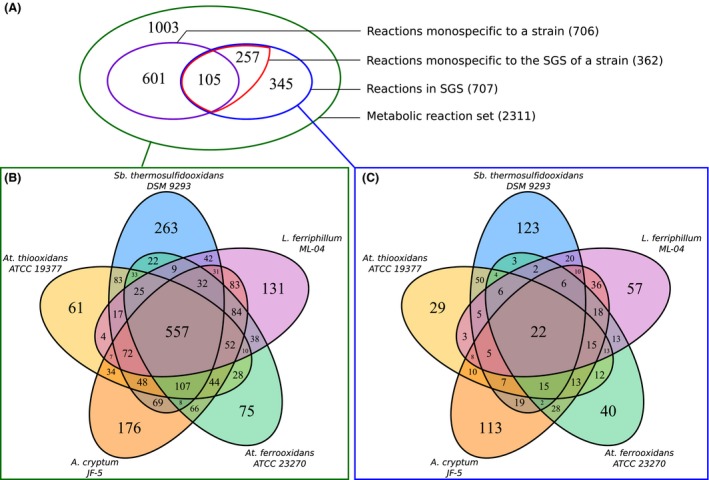
Reactions distribution of the five biomining bacteria. Diagram (A) illustrates how the set of reactions composing the meta‐metabolism is monospecific or multispecific, and also which part of a reaction is involved in *sets from genome segments* (SGS). The Venn diagram (B) illustrates how the set of reactions composing the meta‐metabolism is distributed among bacteria. The Venn diagram (C) illustrates how the set of reactions involved into the SGS is distributed among the bacteria.

*Acidiphilium cryptum*,* Acidithiobacillus ferrooxidans*,* Acidithiobacillus thiooxidans*,* Leptospirillum ferriphilum,* and *Sulfobacillus thermosulfidooxidans* have, respectively, 176, 75, 61, 131, and 263 specific metabolic reactions (from 1470, 1190, 1182, 1194, and 1418 total reactions). Merging the five metabolic networks generates a network composed of 2311 reactions, where 30% of them (706 reactions) are exclusive to single strains (Fig. 2A). Conversely, around 70% (1605 reactions) are conserved in the five genomes, describing a core of common pathways (Fig. 2B). Conserved reactions are related to the generation of precursor metabolites, energy, and basal metabolism. Specific reactions are mostly related to degradation of complex sugars, organic acids and subproducts of protein synthesis, indicating that major metabolic specificities are related to secondary metabolism processes. Noteworthy, these metabolic specificities distinguish two major groups: (1) reactions associated with energy source requirements (mainly iron oxidation) present in autotrophs (*At. ferrooxidans* and *L. ferriphilium*) and chemo‐heterotroph (*Sb. thermosulfidooxidans*), and (2) reactions related to organic degradation compounds of organoheterotrophs (*A. cryptum*) corroborating previous descriptions of specific proteins involved in bioleaching (Baker and Banfield 2003).

### SGS are highly specific to each bacterial strain and represent operons

Overall, SGS involve 707 distinct reactions (30.6 % of the meta‐metabolism). Figure 2A and C show that among them, 362 distinct reactions (51%) are *single strain SGS specific*. This proportion is surprisingly large considering that only 105 of 707 reactions (~15%) are *monospecific* (Fig. 2A and B). In addition, when considered as sets of reactions, most of SGS are *single strain specific* (183 or ~60 % – see third column in Table 1) and almost unique in their strain (4–11% of SGS from a strain share the same set of reactions with another SGS of the same strain).

**Table 1:** Number of *sets from genome segments* (SGS) and the related number of sets of reactions obtained when each SGS is projected onto the metabolic network of the corresponding bacterial strain and the microbial consortium meta‐metabolism. Due to the existence of common reactions to several organisms, the number of reactions within SGS of the consortium is not the sum of the number of reactions of each strain. The comparison of reaction sets was done only by considering the frontier of the SGS: two reaction sets are considered as similar if they have at least one start reaction and one end reaction from their SGS in common. The specific ones are those from distinct organisms that are not similar

Bacteria	Number of distinct SGS	Number of distinct reaction sets	Number of specific reaction sets according to SGS boundaries
*A. cryptum* JF‐5	98	92	45
*At. ferrooxidans* ATCC 23270	61	59	22
*At. thiooxidans* ATCC 19377	67	61	28
*L. ferriphilum* ML‐04	78	72	38
*Sb. thermosulfidooxidans* DSM 9293	92	83	50
Community	396	308	183

*A. cryptum, Acidiphilium cryptum; At. ferrooxidans, Acidithiobacillus ferrooxidans; At. thiooxidans, Acidithiobacillus thiooxidans; L. ferriphilum; Leptosprillum ferriphilum; Sb. thermosulfidooxidans, Sulfobacillus thermosulfidooxidans*.

In parallel, sets of genes emphasized by SGS were compared to predicted operons (Pathway Tools (Karp et al. 2010)) or known operons as stored in ProOpDB (Taboada et al. 2012) and DOOR2 (Mao et al. 2009) databases for *A. cryptum* and *At. ferrooxidans*. A set of SGS gene was considered as an operon when its Jaccard measure is greater than or equal to 0.6, which represents 65.4% of all sets of SGS genes (highlighted as pink segments in Fig. 4). Complementary, and for the sake of support the SGS prediction, SGS genes expression was analyzed. These genes are significantly differentially expressed for others set of genes located in the vicinity; which confirms the SGS functional interest in accordance to available experimental stresses (see File S3 and Appendix S3).

### SGS are complementary to promote metabolic pathways and show putative cooperations

**Figure 3:**
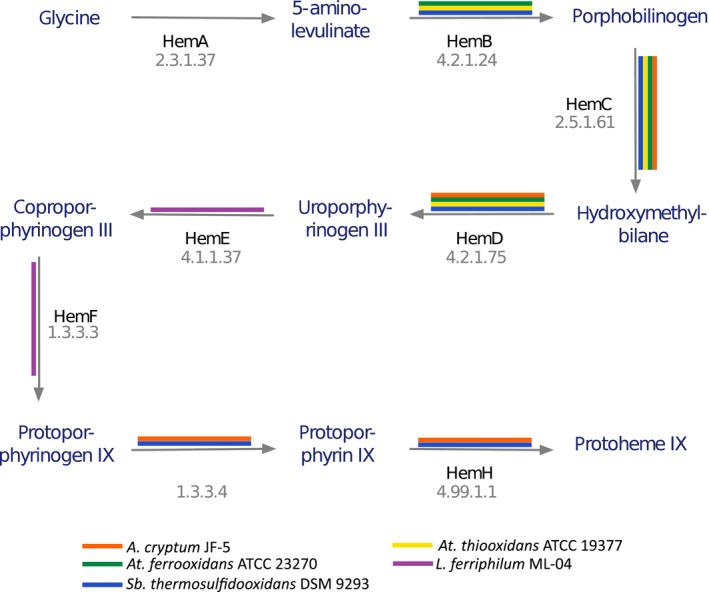
*Superpathway of heme biosynthesis from glycine* from Metacyc (PWY‐5920). Each color edge represents a reaction involved into a *sets from genome segments* (SGS). The orange one is for *Acidithiobacillus cryptum*, the purple one for *Leptospirillum ferriphilum*, the blue one for *Sulfobacillus thermosulfidooxidans*, the yellow one for *Acidithiobacillus thiooxidans,* and the green one for *At. ferrooxidans*.

Figure 3 and Figure S3 show the projection of SGS on metabolic pathways of interest (resp. on Superpathway of heme biosynthesis from glycine and the whole community metabolism) with a particular emphasis on monospecific SGS. While SGS are widely spread over the community metabolic network, it is worth noticing that they remain mostly complementary. Indeed, whereas four SGS are totally disconnected from others, mostly all SGS sets of reactions are separated by at most two reactions (98% of SGS sets of reactions are separated by a gap of one reaction only). In particular, bioleaching pathways are very well covered by distinct SGS (see supplementary materials for details): Superpathway of sulfate assimilation and cysteine biosynthesis (nine SGS), NAD biosynthesis I (from aspartate) (2 SGS), NAD biosynthesis II (from tryptophan) (2 SGS), Superpathway of heme biosynthesis from uroporphyrinogen‐III (4 SGS), heme biosynthesis from uroporphyrinogen‐III I (3 SGS), heme biosynthesis from uroporphyrinogen‐III II (4 SGS), Superpathway of heme biosynthesis from glutamate (8 SGS), Superpathway of heme biosynthesis from glycine (8 SGS), and glutathione biosynthesis (2 SGS).

**Figure 4:**
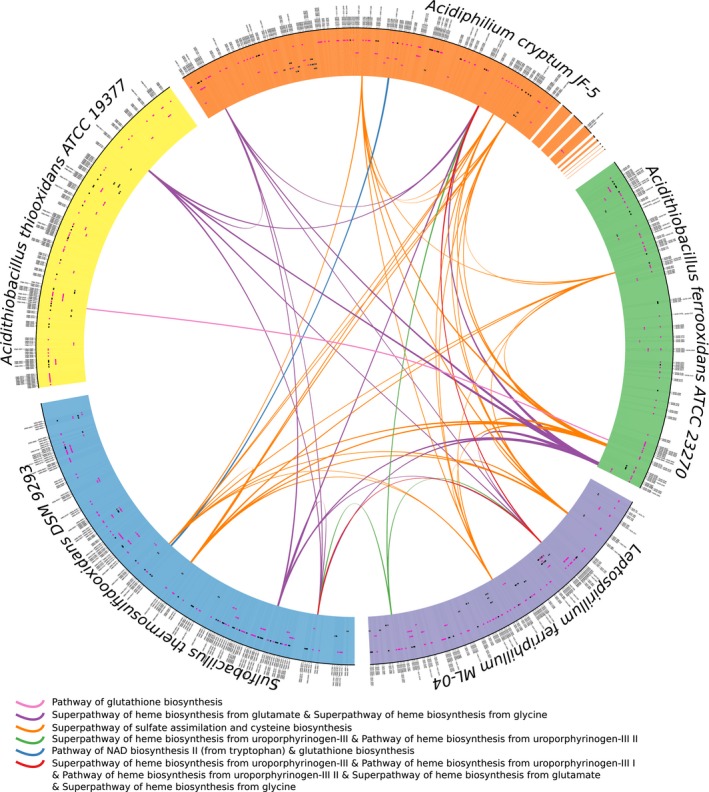
Metabolic copper bioleaching relationships between *sets from genome segments* (SGS) at the metagenomic scale. The outside bands represent the five bacterial genomes. The blue, purple, green, orange and yellow bands refer, respectively, to *Sulfobacillus thermosulfidooxidans*,* Leptospirillum ferriphilum*,* Acidithiobacillus ferrooxidans*,* Acidiphilium cryptum,* and *Acidithiobacillus thiooxidans* genomes. The black and pink segments over the genomes illustrate the SGS, where the pink ones are SGS similar to operons whereas the black ones are those that are not similar to operons. Gray parts of the segments indicate genes that do not participate in the meta‐metabolic scale. A link connecting two SGS indicates that those two SGS participate in the same pathway. The color of the link is specific to a set of pathways.

Interestingly, no single bacterial strain is able, alone, to execute a whole pathway related to bioleaching, but rather a cooperation of SGS between different strains is necessary. For illustration, Figure 4 pinpoints putative collaborations of each bacteria to bioleaching pathways via their respective SGS. Each ribbon connects SGS of the five bacteria that participate in the same pathway (one ribbon color per group of bioleaching pathways). Precisely, the bacterial participation is as follows: *A. cryptum* has six linked SGS, whereas *At. ferrooxidans* and *Sb. thermosulfidooxidans* have five each, *L. ferriphilum* has four and *At. thiooxidans* has two. Notably these SGS are homologous (see Fig. S10 and the SGS list). Similarly, Figure 4 (blue) shows a putative homologous collaboration between one SGS from *A. cryptum* and one from *Sb. thermosulfidooxidans* in the NAD biosynthesis I (from aspartate) pathway (see Fig. S5). Contrarily, the red link presents a chaining of two SGS (one from *A. cryptum* and the other one from *L. ferriphilum*) into the five pathways of the heme biosynthesis (the orange and purple segments in Fig. S6–10). The combination of duplicated SGS across bacteria and chaining of SGS is also observed. The purple links depict the putative collaborative participation of five SGS into the Superpathway of heme biosynthesis from glutamate but also the Superpathway of heme biosynthesis from glycine. These superpathways are variants but they share the same reactions. In this pathway, the SGS from *A. cryptum* and *Sb. thermosulfidooxidans*, but also those from *At. ferrooxidans* and *At. Thiooxidans,* are similar and participate into a chain with the SGS of *L. ferriphilum* (see Fig. 3 and Fig. S10).

### Transporters and common goods as consequence of SGS combination

Assuming that SGS from different species decipher putative intricate collaborations of microbial strains at the community level, metabolites surrounded by two SGS from different species represent a potential common good that must be, respectively, imported and exported from bacterial cytoplasm via transporters. Following this assumption, APS (adenosine phosphosulfate), PAPS (phosphoadenosine phosphosulfate), protoporphyrinogen, uroporphyrinogen‐III, and hydrogen peroxide are theoretical common goods, because they connect SGS reactions for bioleaching pathways. Except hydrogen peroxide these compounds are metabolic intermediates that participate mainly in heme biosynthesis and amino acid synthesis processes (Valdés et al. 2003). While, there is no currently experimental evidence for proteins involved in the direct mobilization of these metabolites inside or outside bacterial organisms, interestingly we identified classical transporters like ATP Binding Cassette (ABC), Resistance Nodulation Division (RND), and Major Facilitator Superfamily (MFS) systems encoded next to SGS involved in sulfate assimilation, serine biosynthesis, and mainly heme pathways. These results pinpoint the already known central role of heme to control the whole bioleaching process (Valdés et al. 2003), whereas APS, PAPS, protoporphyrinogen, and uroporphyrinogen‐III may play an indirect role via their modulation of concentrations that potentially impact heme biosynthesis. Interestingly, one must notice that these common good metabolites connect SGS from *L. ferriphilum* on one hand to the SGS from all four other strains on the other hand.

## Discussion

### Generic paradigm for genome‐wide integrative study for a bacterial community

This study integrates genomic and metabolic knowledge to study a reduced microbial community. By using a simple parsimony assumption on topological knowledge (i.e., genome organization and metabolic network), the SGS paradigm proposes a genome‐wide description of functional units that are necessary to achieve a given function hold by a microbial community. This approach could be considered as a promising alternative to microbial cross‐feeding analysis when quantitative experiments are not feasible. In particular, one considers SGS technique as preliminary to recent Flux Balance like techniques that need to be considered as an objective function or quantitative features for each strain.

These limitations are particularly true for extremophiles strains for which biological knowledge is sparse. Indeed, whereas genome sequencing and assembly techniques are trustworthy for extremophiles, it remains difficult to consider an exhaustive definition of metabolic networks, that are mostly incomplete (McCloskey et al. 2013) or misannotated (Liberal and Pinney 2013). To overcome this weakness, our SGS technique tolerates uncertainties, via a flexible constraints‐based implementation. For instance, one considers genes within SGS that are not directly related to the metabolic pathways of interest or even not depicted as catalytic genes (c.f. gray genes in Fig. 1).

To further confirm this methodological advantage, we advocate for complementary application studies. In particular, SGS applications are of potential interest for analyzing metagenomic or meta‐transcriptomic results that investigate, as observed in nature, more complex microbial natural communities. Thus, beyond a further validation of SGS, such a extensive application could be of potential use to give insights about microbial richness and its relation to metabolic pathways use and/or biogeochemical processes of interest.

### Compartments are the consequence of genome organization

Interestingly, the bioleaching community shares most of the reactions involved in the analyzed pathways (Fig. 2B), which supports the idea of a metabolic redundancy within the community that usually promotes the use of a “single‐cell assumption” to investigate meta‐genome experiments. All these shared reactions are part of the core of conserved bacterial pathways present in most strains described to date (Lapierre and Gogarten 2009), indicating that conservation of the pathways is a general feature of bacterial organisms and not a particular property of the bioleaching community. However, despite this high redundancy of metabolic reactions, SGS are not evenly distributed between bacteria of interest (Fig. 2C). SGS mostly covered functional units known as operons. As accepted, the conservation of a gene in an operon is a strong parameter of functional neighborhood inferences, where essential genes are more clustered than the average genes (Nuñez, 2013). Few SGS or functional units are replicated between all bacterial strains, even between pairs of bacteria, which implies that, overall, functional units at the community level are specialized to each strain. When projected on the whole bioleaching metabolic pathways, SGS are mostly complementary. This point shows, at a metagenomic level, evidences that genome organization could play a role to explain cross‐feedings between microbial strains at the community level. Furthermore, the bacterial strain‐specific SGS distribution characterizes a *functional compartmentalization* that is, again, a direct consequence of a simple parsimony assumption on genome and metabolic knowledge integration. Complementary, no single bacterium is able alone, to monitor the whole copper bioleaching network (Fig. 3 and Fig. S3), which clearly indicates a specialization of the strains. From an evolutionary point of view, this compartmentalization reflects the structured evolution of the five genomes and their corresponding metabolisms. Compartments could be also considered as a way to contain all toxicities that are enhanced by the whole copper bioleaching. In particular, the mining community is composed of extremophiles species that are already known to handle severe and distinct environmental stresses. By promoting bacterial diversity rather than the presence of a single ubiquitous bacterial strain that handles the whole bioleaching, one can assume that the community systems share all stresses based on respective strain capacity while maintaining an overall oxidation process that is chemically and energetically optimal for the whole. From a systems biology point of view, this emergence of functional compartments represents another insight that promotes the concept of modularity for improving the stability of the whole system as previously observed at the single‐cell metabolic scale (Kitano 2004; Larhlimi et al. 2011), but herein at the community level. Finally, from a biomining viewpoint, this clearly advocates for the need for considering bacterial compartments and their interactions and confirms, with no a priori, the lack of interest in studying a single bacterial specie like previously *At. ferrooxidans* to embrace the whole bioleaching process.

### Putative community common goods

When following a given metabolic pathway, one emphasizes connections between different SGS that belong to different bacterial strains (see Fig. 1 and Fig. 3), supporting the concept that functional interactions between members are crucial (Baker and Banfield 2003). Interestingly, the six bioleaching superpathways are all functionally interconnected via SGS. More precisely, SGS are connected either because they are functionally redundant (e.g., superposed colors in Fig. 3 and Figure S3) – or complementary on metabolic pathways (e.g., a succession of colors in Fig. 3 and Figure S3). In particular, *Sb*. *thermosulfidooxidans* and *A*. *cryptum* share most of the SGS. Interestingly, besides its elevated connectivity, *A*. *cryptum* shows the largest number of SGS related with NAD(H) biosynthesis metabolic pathway, and this despite not considering NAD(H) molecule compound for connectivity purpose (see Method section). Such connections between all five bacterial strains by SGS related to NAD(H) superpathways confirms that bioleaching requires the reduction potential given by the NAD(H) molecule, denoting its multispecificity participation in bioleaching, as well as an elevated robustness to external factors (Yus et al. 2009).

Previous studies also correlate the capability of the community to bioleach copper with the ability to generate biomass, a process that requires NAD potential for its realization (Valdés et al. 2010). Because most of NAD(H) SGS are monitored by *A. cryptum*, this strain plays an unexpected but major role in structuring the bioleaching community, indicating that the collaboration inside the community lies principally in its ability to complement different metabolic functions, as spread between the five strains.

Finally, such collaborations highlight putative transporters between bacterial strains, as well as the common good metabolites shared by two species in the bioleaching context. The analysis of these metabolites surrounded by two SGS indicates potential transporters to seek within genomes, but also confirms the main interest of heme for monitoring bioleaching at the community scale. Indeed SGS analysis advocates that APS, PAPS, protoporphyrinogen and uroporphyrinogen‐III are potential regulators of heme pathway. Other transporters, localized next to some SGS, were identified related to serine production and sulfate assimilation. Interestingly, it has been described that in *At. ferrooxidans* these two processes are directly involved in cysteine production and Fe‐S cluster formation, two crucial molecules highly required during the bioleaching of copper (Valdés et al. 2003). The genomic proximity between SGS and transporters strongly suggests that strain‐specific SGS can interact with other organisms of the consortia through the biosynthesis and transport of common metabolic goods, in this case mainly related with heme and sulfur assimilation pathways. Beyond the bioleaching application and speculative interpretations, SGS results provide functional enrichment to metagenomic knowledge as well as guidelines for future molecular investigations at the community scale, in particular in the search and identification of putative transporters necessary for a cooperative metabolic behavior at the metabolic scale, especially when cross‐feeding experiments are not feasible.

## Data Archiving

All data and programs are available in: http://philippe.bordron.net/sgs-at-the-community-scale.html. Program available via a web application: http://mobyle.inria.cl/. Supplementary information is available at Journal's website.

## Conflict of Interest

None declared.

## Supporting information


**Appendix S1.** Definition.

**Appendix S2.** Choosing SGS parameters.

**Appendix S3.** Transcriptomic insights.

**Appendix S4.** Coexpression in SGS.

**Figure S3.** Projection of reaction sets corresponding to SGS on the meta‐metabolic network.

**Figure S4.** Superpathway of sulfate assimilation and cysteine biosynthesis from Metacyc (SULFATE‐CYS‐PWY).

**Figure S5.** Pathway of NAD biosynthesis I (from aspartate) from Metacyc (PYRIDNUCSYN‐PWY).

**Figure S6**. Pathway of NAD biosynthesis II (from tryptophan) from Metacyc (NADSYN‐PWY).

**Figure S7.** Superpathway of heme biosynthesis from uroporphyrinogen‐III from Metacyc (PWY0‐1415).

**Figure S8.** Pathway of heme biosynthesis from uroporphyrinogen‐III I from Metacyc (HEME‐BIOSYNTHESIS‐II)

**Figure S9.** Pathway of heme biosynthesis from uroporphyrinogen‐III II from Metacyc (HEMESYN2‐PWY).

**Figure S10.** Superpathway of heme biosynthesis from glutamate from Metacyc (PWY‐5918).

**Figure S11.** Pathway of glutathione biosynthesis from Metacyc (GLUTATHIONESYN‐PWY).

**Data S1.** The file SpplementarymaterialsS1.xls contains the list of orthologs between the *At. ferrooxidans* ATCC23270 and *At. ferrooxidans* Wenelen strains.

**Data S2.** The file SpplementarymaterialsS2.xls contains the complete list of SGS.

**Data S3.** The file SpplementarymaterialsS3.xls contains the list of SGS involved in the bioleaching pathways.
