# Supplementary material for: Putative bacterial interactions from metagenomic knowledge with an integrative systems ecology approach
Source: Microbiologyopen. 2015 Dec 17;5(1):106–17. doi: 10.1002/mbo3.315 (PMC4767419; doi:10.1002/mbo3.315)
Supplement: Supplementary file 1 — Appendix S1. Definition. [file MBO3-5-106-s001.pdf]

Definition

The SGS problem

In order to define the SGS problem, the following definitions are required.

Definition 1 (Genome segment). *Given a circular sequence of genes $\mathcal{G} = g_1 \dots g_n$, we define a genome segment of $\mathcal{G}$ as a sequence $\sigma = g_i \dots g_j$, where $1 \leq i \leq j \leq n$, or a sequence $\sigma = g_i \dots g_n g_1 \dots g_j$, where $0 \leq j \leq i \leq n$.*

Definition 2 (Induced subgraph). *Let $\mathcal{G}$ be a circular sequence of genes and (R, E) the reaction graph associated to such sequence. Given a genome segment σ of $\mathcal{G}$, the induced subgraph is (R_σ, E_σ) where $R_\sigma = \{r \in R \,|\, \exists\ g \in \sigma,\ r \in catalyze(g)\}$ and $E_\sigma = \{(r, r') \in E \,|\, \exists\ g, g' \in \sigma,\ r \in catalyze(g),\ r' \in catalyze(g')\}$.*

The induced subgraph reflects the regulatory influence of the genes in the segment, their corresponding enzymes products and the catalyzed reactions relation of these enzymes.

Definition 3 (Useful reaction set). *Given a reaction graph H, a start reaction r and a target reaction r', the useful reaction set from H is the set of reactions that appear in at least one of the elementary paths from r' to r' in H.*

Now we can define the *Set from Genome Segment* (SGS) problem as follows. This definition is a little bit different from the *Shortest Genome Segment* from Bordron *et al.*, 2013 but is morally the same as we found after re-working the notion that this new definition is more adequate.

Definition 4 (Set from Genome Segment problem). *The SGS receives the following data: a genome sequence $\mathcal{G}$, a reaction graph (R, E), a map $catalyze : G \to \mathcal{P}(R)$ and two reactions $r, r' \in R$.*

A solution to a SGS problem is the set of genes τ that is contained into the genome segment σ of $\mathcal{G}$ such that the genes of τ catalyze the reactions of the useful reaction set from r to r' in the induced subgraph (R_σ, E_σ).

A solution to a SGS problem points to a set of genes that take an active part in the metabolic regulation and form an active gene unit. Among all possible SGS, we are interested in those that follow a simple parsimony assumption: one seeks for the small genome segments that jointly point for consecutive reactions when projected onto the metabolic network. To emphasize these particular ones, one considers a start reaction r_s and a target reaction r_t that can be catalyzed by enzymes associated to genes with at most a gap of 10 genes between them and SGS as the set of genes contained into the segment of genes composed at most of 20 genes.

Selection of dense SGS

Among the huge number of resulting SGS, only SGS with a functional meaning were selected. As proposed in (Bordron *et al*, 2011) a complementary feature called genomic density was considered to emphasize SGS that can be assumed as functional units or operons.

Definition 5 (Genomic density). *Let be τ a SGS and σ the smaller segment containing τ. The genomic density d_G of τ is defined as follow:*

$$d_G(\tau) = \frac{|\tau|}{|\sigma|}$$

The genomic density takes values in $]0, 1]$. Intuitively, the higher the density is, the more the genes of τ are grouped together on the genome and take part of the induced reaction chains. A genomic density equal to 1 indicates that all the genomic elements of τ are consecutive on the genome and take part of the metabolic pathway. A genomic density near 0 indicates that at least one element of τ is distant from the others on the genome. Following previous results (Bordron *et al*, 2011) we fixed the genomic density threshold δ at 0.6, above which selected SGS have been shown mainly as operon. This allows to reduce the number of SGS of interest by considering the denser ones without losing information.

Dominant SGS

A SGS can be included into other SGSs. In order to reduce the number of SGSs by selecting those that are interesting, we define then the notion of domination between SGSs.

Definition 6 (Domination). *A SGS τ dominates another SGS τ' if and only if τ' is include in τ and $d_G(\tau) \geq d_G(\tau')$.*

Definition 7 (Useful Reaction Set*). *As a dominated SGS can involve some reactions that do not appear in the useful reaction set of the dominant one, we define a new reaction set that is the union the useful reaction set of the dominant SGS with the useful reaction set of the SGS it dominates.*

Definition 8 (Start and end reaction sets)**.** *Moreover, the set of start reactions, and the set of target reactions from dominated SGS are also group respectively with the start and stop reactions of the dominant SGS. Distinct couples of reactions can produce the same segment. Dominant SGS also drop the information about those couples.*
