# Supplementary material for: Putative bacterial interactions from metagenomic knowledge with an integrative systems ecology approach
Source: Microbiologyopen. 2015 Dec 17;5(1):106–17. doi: 10.1002/mbo3.315 (PMC4767419; doi:10.1002/mbo3.315)
Supplement: Supplementary file 2 — Appendix S2. Choosing SGS parameters. [file MBO3-5-106-s002.pdf]

Choosing SGS parameters

The section is about choosing meaningful parameters for SGS calculation. We use the well-known *E. coli* bacteria as model organism. First we estimate the SGS length parameter by looking at the size of operons and regulons in *E. coli* genome. Then, we set the SGS density parameter by looking at the operonic and regulonic relevance of SGS in function of the density of the SGS. Finally, we choose the maximum query distance of a SGS, which is the maximum genomic distance between the source and target reactions, by looking at how much relevant are SGS depending of the query distance.

SGS maximum length parameter

Firstly, we take a look at the metabolic operons and metabolic regulons of *E. coli.* We use data from RegulonDB 8.7 for this purpose. Operons are directly obtained from the RegulonDB files, and regulons are reconstructed by merging the transcription units that have the same set of transcription factors as the regulon definition states.

From the 2638 operons listed in RegulonDB, 2554 (96.9%) and 2625 (99.5%) of them have respectively at most 5 genes and at most 10 genes (see the red histogram in Figure i.A). The longest operon is composed of 16 genes. When considering the metabolic genes, only 1042 (39.5%) and 336 (12.7%) of them contain respectively at least 1 metabolic gene and at least 2 metabolic genes (see the red histogram in Figure i.B). An operon contains a most 13 metabolic genes.

From the 483 regulons, 473 (95.03%) and 459 (97.9%) of them have respectively at most 10 genes and at most 20 genes (see the blue histogram in Figure i.A). The biggest regulon contains 66 genes. When considering the metabolic genes, only 332 (68.7%) and 201 (41.6%) of them contain respectively at least 1 metabolic gene and at least 2 metabolic genes (see the blue histogram in Figure i.B). A regulon contains a most 21 metabolic genes.

Note that, at the difference of the genes of an operon, genes of a regulon are not consecutive on the genome. From the previous observations, we fix that SGS must have at most a length of 20 genes. With this value, we have chance to cover at least all the operons, but also a big part of regulons if they have genes close to each other on the genome.

SGS density parameter

Secondly, from the data of Ecocyc 18.0, we compute the set of (dominant) SGS that have a maximum length of 200 genes for all the couple of reactions that are catalyzed by genes distant of at most 200 genes. We obtain a set of 710 SGS. In order to compare each SGS with operons and regulons, we define the *operonic relevance* and the *regulonic relevance* of a SGS.

Operonic relevance

We define the similarity of a SGS S with an operon O by using the Jaccard measure that is defined as follow:

$$\text{Jaccard}(S, O) = \frac{|S \cap O|}{|S \cup O|}$$

The Jaccard measure is also called the mutual coverage measure. It takes value in $[0, 1]$. The higher the value is, the more similar are S and O.

By keeping the maximum similarity value obtains when comparing a SGS with each operon from $\mathcal{O}$ which is the set of known operons, it is then possible to define the *operonic relevance* of the SGS.

$$R_{\mathcal{O}}(S) = \max_{O \in \mathcal{O}} \{\text{Jaccard}(S, O)\}$$

Regulonic relevance

We use the *cover measure* instead of the Jaccard measure the measure the regulonic relevance of a SGS. For a given SGS and a given regulon, we note S and R respectively their set of genes. The cover measure is defined as follow.

$$\text{Cover}(S, R) = \frac{|S \cap R|}{|S|}$$

The cover measure takes value between 0 and 1. The higher the value is, the bigger the part of A is include in B. For the set of known operon $\mathcal{R}$, the *regulonic relevance* $R_{\mathcal{R}}$ of the SGS S defines the maximum proportion of S which is include in a regulon from $\mathcal{R}$.

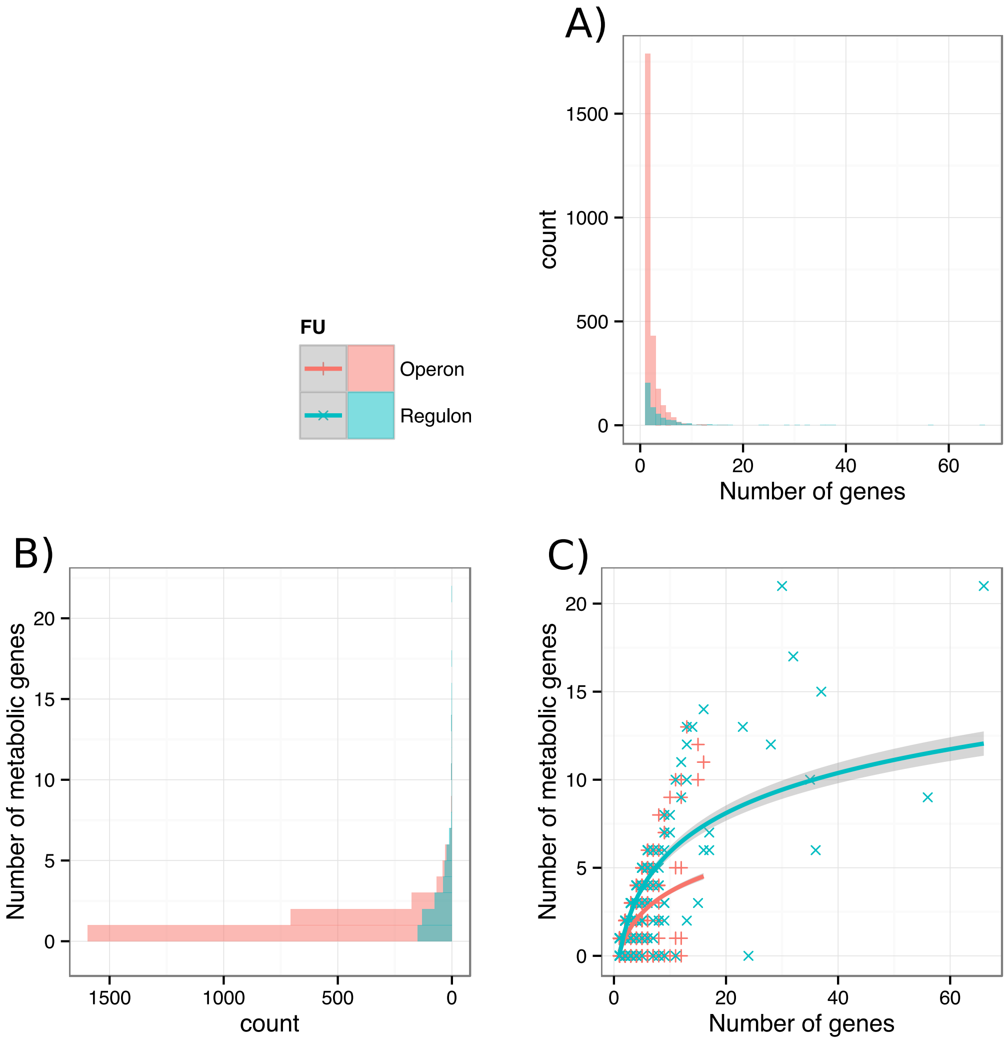

Figure i: Number of genes in operons (in red) and regulons (in blue). A) Distribution of the number of genes composing operons and regulons. B) Distribution of the number of metabolic genes composing operons and regulons. C) Scatter plot presenting the relationship between the number of genes and the number of metabolic genes in operons and regulons. The red curve and the blue curve present respectively the regression of the number of genes dispersion for the set of operons and the set of regulons. For each regression, the grey part represents the confidence interval (at 95%).

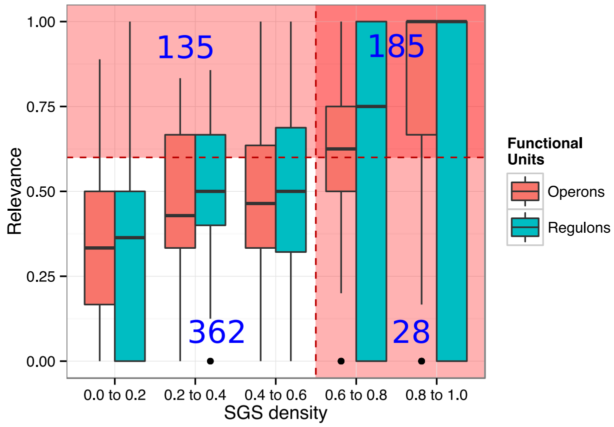

Figure ii: Operonic relevance (in red) and Regulonic relevance (in blue) of SGS in function of their density. The vertical dotted line represents the density threshold which is set to 0.6. The horizontal dotted line represents the relevance selection threshold which is also set to 0.6. Each number in the four parts of the boxplot is the number of SGS presents in this part.

$$R_{\mathcal{R}}(S) = \max_{R \in \mathcal{R}} \{\mathrm{Cover}(S, R)\}$$

The Figure ii presents the operonic and regulonic relevance of SGS according to their density. This figure shows that most of SGS (185 from 213 to be precise) with a density higher or equal to 0.6 are relevant from an operonic or regulonic point of view (they have a relevance score higher or equal than 0.6).

In addition to this analysis, 212 from the 213 SGS with a genomic density greater or equal to 0.6 have a length of at most 20 genes (see figure Figure iii.B). The last one is 25 genes long but with an operonic relevance of 0.565 and a regulonic relevance of 0.125 and thus not selected as relevant from an operonic and regulonic viewpoint

We chose 0.6 as the density threshold from this observation.

SGS maximal query distance parameter

Finally, we define the set of SGS queries, which is the set of couples of reactions that will be used to compute SGS, by looking at the genomic distance that exists between the source and target reaction. We look at how much SGS with a density greater of equal to 0.6 are conserved when selecting couples of reactions according to their genomic distance (via the enzymes that catalyze them). The Figure iii summarizes the SGS distribution when considering the density, length and query distance of SGS. From the set of 213 SGS with a density greater of equal to 0.6, 211 of them have a query distance of at most 9 (see Figure iii.F for details). The query distance of the two others is 17, with one of them with an operonic relevance of 0.565 and a regulonic relevance of 0.125 and the other one with an operonic relevance of 0.6 and a regulonic relevance of 0.133.

When the maximum query distance is set to 10, the amount of non-relevant SGS we have computed is limited. Indeed, by setting the SGS maximum length to 20 and the maximum query distance to 10, we obtain a set of 247 SGS instead of a set of 710 SGS (see Figure iii.E for details) where 211 of them are relevant from an operonic and regulonic viewpoint (in practice, we only lost one interesting SGS).

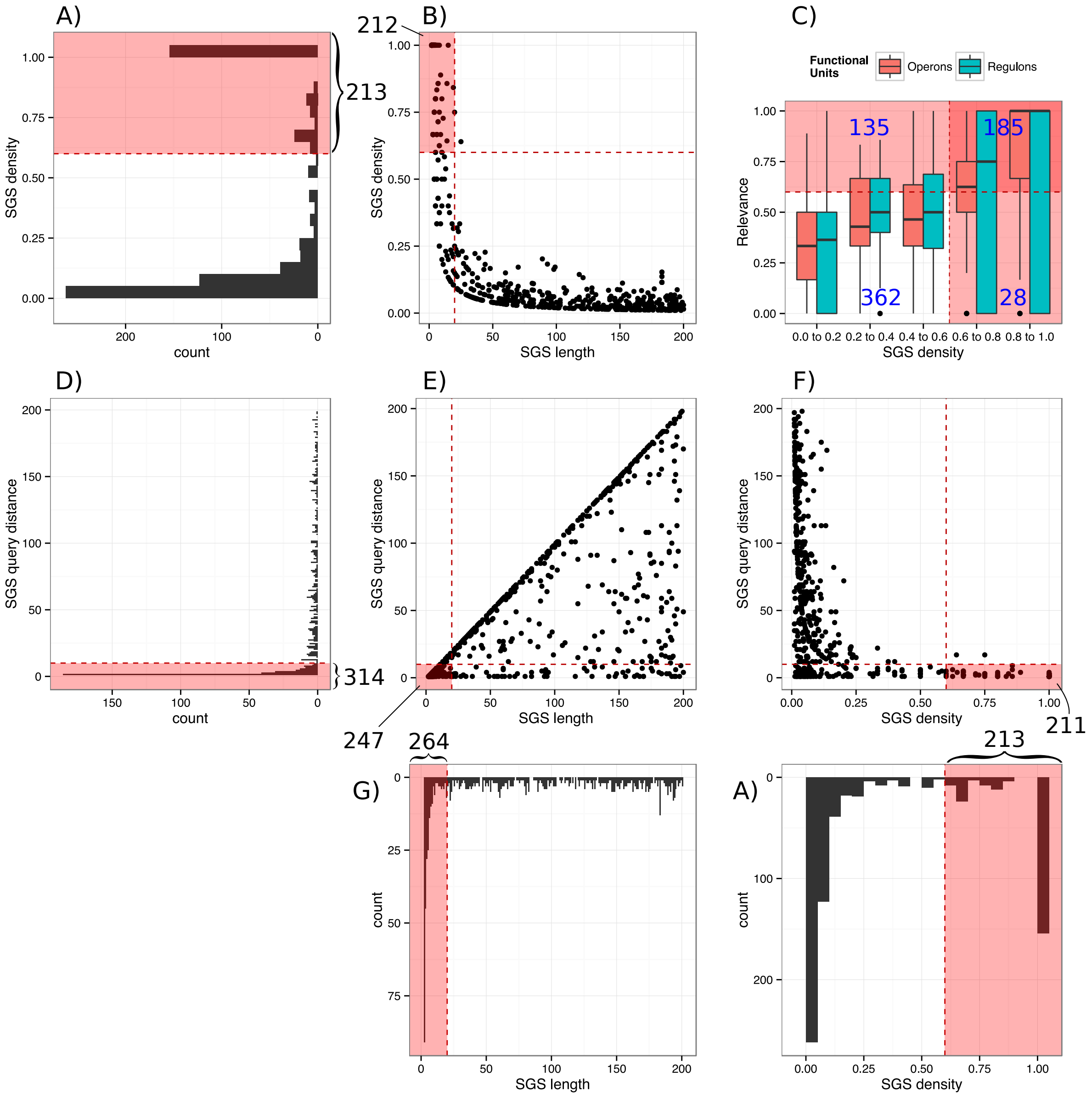

Figure iii: SGS distribution according to the length, query distance and density parameters. A) Frequency of SGS when looking at their density. B) Relationship between length of SGS and density of SGS. C) Operonic relevance (in red) and Regulonic relevance (in blue) of SGS in function of their density. D) Frequency of SGS when looking at their query distance. E) Relationship distribution between length of SGS and query distance of SGS. F) Relationship between density of SGS and query distance. G) Frequency of SGS when looking at their length. The red part in each figure presents the selected SGS when the length is at most 20, the query distance is at most 10 and the density is at least 0.6.
