# Supplementary material for: Putative bacterial interactions from metagenomic knowledge with an integrative systems ecology approach
Source: Microbiologyopen. 2015 Dec 17;5(1):106–17. doi: 10.1002/mbo3.315 (PMC4767419; doi:10.1002/mbo3.315)
Supplement: Supplementary file 3 — Appendix S3. Transcriptomic insights. [file MBO3-5-106-s003.pdf]

Transcriptomic insights

For the sake of illustration, we project available transcriptomic information for *At. ferrooxidans SGS*. Because of the lack of knowledge about the whole community, this companion information was solely considered as supplementary.

At. ferrooxidans transcriptome

In addition to the public genomic and metabolic information, we use, as a transcriptomic enrichment, a set of dedicated microarray experiments performed on cultures of At. ferrooxidans strain Wenelen (DSM 16786) in BioSigma Reference Laboratory (Colina, RM, Chile). This set of microarray data is accessible through the http://wenelenarray.cmm.uchile.cl website. From a transcriptomic viewpoint, ATCC23270 and Wenelen strains are identical (Levican et al, 2008), especially in bioleaching related studies (see ortholog list between both strains in supplementary materials). Transcriptomic experiments use gene expression in ferric medium as a reference and measure respectively the differential gene expression in (i) shift to sulfur, that is, ferric medium with last minute addition of sulfur, (ii) shift to Chalcopyrite ($CuFeS_2$), (iii) shift to Pyrite (FeS_2), and (iv) shift to quartz (SiO_2) (see supplementary for details).

Results

In order to asset putative interactions between microbial strains via there respective SGS, SGS genes must be specific to each pathway they belong to. For the sake of validation, transcriptomic behaviors of *At. ferrooxidans* SGS genes were analyzed when they are challenged by distinct bioleaching related stresses. For a given SGS with *n* genes, its gene expression was compared

respectively with the set of the *n* preceding (5') and successive (3') genes. In Figure S13, no experience is performed to stress genes related to the heme biosynthesis from glutamate and from the glycine, but rather stresses related to sulfur, iron and copper media. SGS related to heme biosynthesis pathways (purple boxplots) show no difference with their surrounding gene intervals. In addition, SGS related to the sulfate assimilation and cysteine biosynthesis (orange boxplots) show differential behaviors than their surroundings, as well as SGS related to glutathione biosynthesis, emphasizing the metabolic specificity of SGS. Noteworthy, SGS from a same pathways have the same behavior when we look at the expression values.

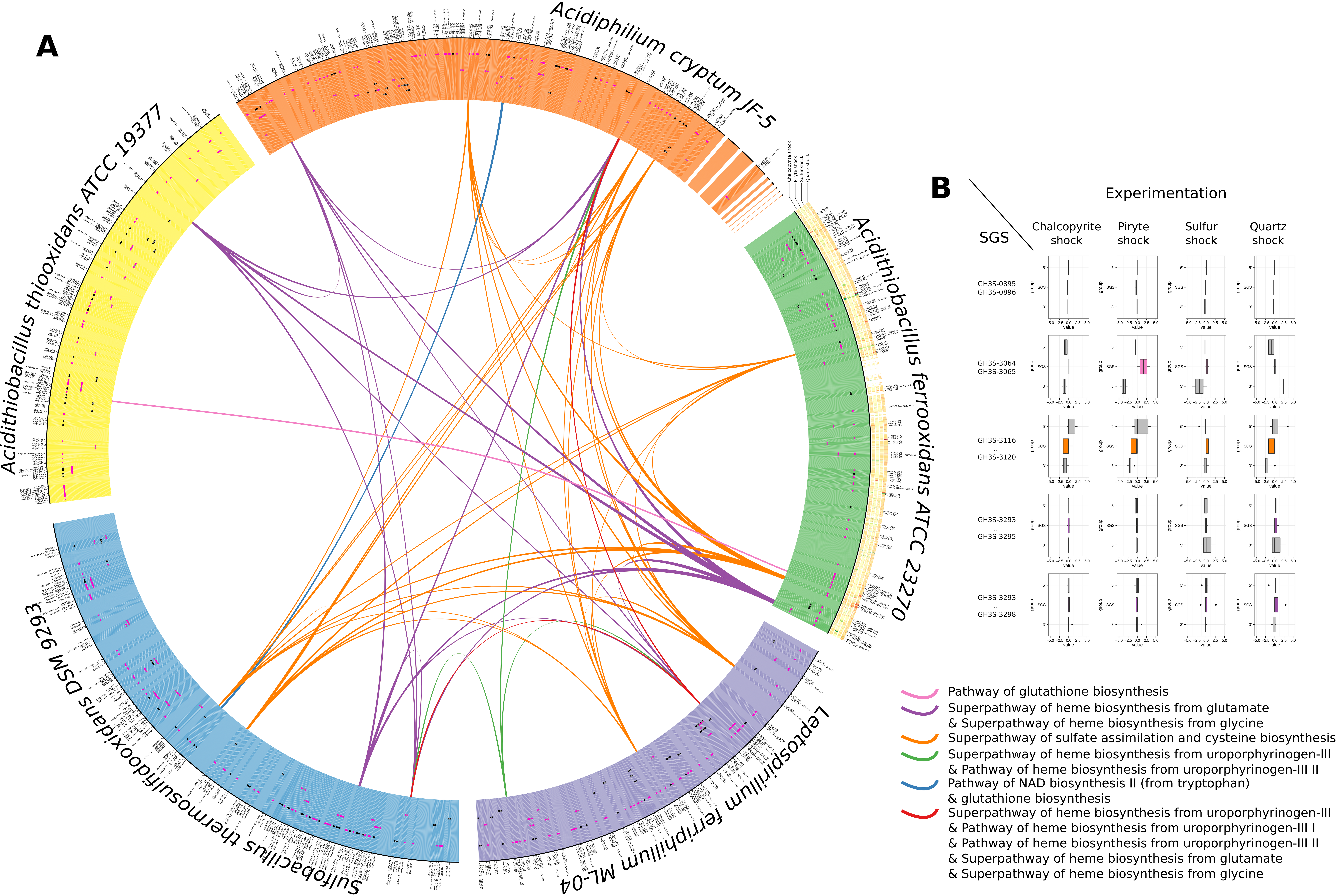
A
Acidiphilium cryptum JF-5
Acidithiobacillus ferrooxidans ATCC 23270
Leptospirillum ferriphilum ML-04
Sulfobacillus thermosulfidooxidans DSM 9293
Acidithiobacillus thiooxidans ATCC 19377
Chalcopyrite shock
Piryte shock
Sulfur shock
Quartz shock
B
Experimentation
SGS
Chalcopyrite shock
Piryte shock
Sulfur shock
Quartz shock
GH3S-0895
GH3S-0896
GH3S-3064
GH3S-3065
GH3S-3116
...
GH3S-3120
GH3S-3293
...
GH3S-3295
GH3S-3293
...
GH3S-3298
Pathway of glutathione biosynthesis
Superpathway of heme biosynthesis from glutamate
& Superpathway of heme biosynthesis from glycine
Superpathway of sulfate assimilation and cysteine biosynthesis
Superpathway of heme biosynthesis from uroporphyrinogen-III
& Pathway of heme biosynthesis from uroporphyrinogen-III II
Pathway of NAD biosynthesis II (from tryptophan)
& glutathione biosynthesis
Superpathway of heme biosynthesis from uroporphyrinogen-III
& Pathway of heme biosynthesis from uroporphyrinogen-III I
& Pathway of heme biosynthesis from uroporphyrinogen-III II
& Superpathway of heme biosynthesis from glutamate
& Superpathway of heme biosynthesis from glycine

Figure S12: Metabolic copper bioleaching relationships between SGS at the metagenomic scale. In the circos representation **A**, the outside bands represent the five bacterial genomes. The blue, purple, green, orange and yellow bands refer respectively to *Sb. thermosulfidooxidans*, *L. ferriphilum*, *At. ferroxidans*, *A. cryptum* and *At. thiooxidans* genomes. The black segments over the genomes illustrate the SGS, where grey parts indicate genes that do not participate in the meta-metabolic scale. A link connecting two SGS indicates that those two SGS participate in the same pathway. The color of the link is specific to a set of pathways. The outer bands of the At. ferrooxidans genome represent the transcriptomic information obtained under stresses appending in the natural biomining environment (red color indicates an under-expression and green color an over-expression). The boxplot matrix in **B** zooms at the particular SGS and their genomic neighbourhood from At. ferrooxidans involved into bioleaching pathways. Colors used inside the boxplot are the same as the link colors interconnecting SGS together.
