# Supplementary material for: Putative bacterial interactions from metagenomic knowledge with an integrative systems ecology approach
Source: Microbiologyopen. 2015 Dec 17;5(1):106–17. doi: 10.1002/mbo3.315 (PMC4767419; doi:10.1002/mbo3.315)
Supplement: Supplementary file 4 — Appendix S4. Coexpression in SGS. [file MBO3-5-106-s004.pdf]

Coexpression in SGS

For ρ a given a group of n genes, we note ρ_i the expression value of the ith gene in ρ and $\overline{\rho}$ the average gene expression in the SGS. An estimation of the variance of the gene expression of ρ is calculated with the following formula:

$$\mathrm{V}(\rho) = \frac{1}{n-1} \sum_{i=1}^{n} (\rho_i - \overline{\rho})^2$$

Random set of genes

A random set of k genes from a genome composed of n genes is obtained by drawing without replacement and in an equiprobable way k genes from the genome.

Random set of contiguous genes

A random set of k contiguous genes from a genome composed of n genes is obtained by drawing in an equiprobable way one gene from the genome and taking the $k-1$ next genes in the circular bacterial genome.

Equivalent set of random set of (contiguous) genes

Given a set $S = \{s_1, s_2, \dots, s_m\}$ of m sets of genes, the set $S' = \{s'_1, s'_2, \dots, s'_m\}$ is an equivalent set of S such as each s'_i is a random (contiguous) set of genes of the size $|s_i|$ from the genome for each $s_i \in S, 1 \leq i \leq m$.

The Figure S13 on the next page presents the comparison in *At. ferrooxidans* between distribution of the variance of the SGS, the distribution of variance of 1000 equivalent sets of random group of genes, and the distribution of variance of 1000 equivalent sets of random group of contiguous genes.

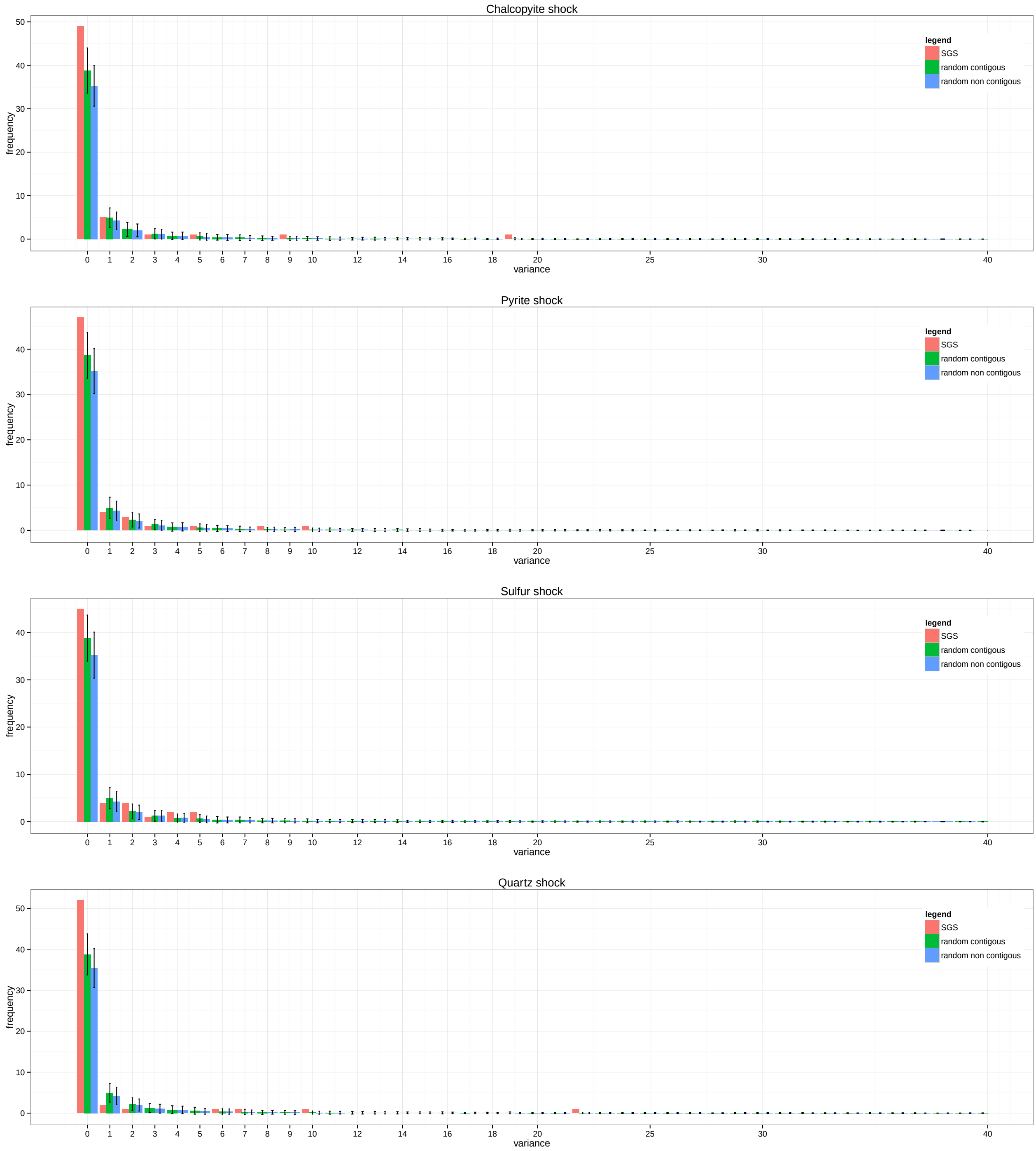

Figure S13: Distribution of variance of SGS expressions (in red) compared to the average distribution of variance of 1000 equivalent sets of random sets of contiguous gene expressions (in green) and the average distribution of variance of 1000 equivalent sets of random sets of gene expressions (in blue) in four dinstinct environmental conditions : chalcopyite shock, pyrite shock, sulfur shock and quartz shock. The whiskers on each frequancy of the random distributions represent the standard deviation for the given frequency.
