# Supplementary material for: Putative bacterial interactions from metagenomic knowledge with an integrative systems ecology approach
Source: Microbiologyopen. 2015 Dec 17;5(1):106–17. doi: 10.1002/mbo3.315 (PMC4767419; doi:10.1002/mbo3.315)
Supplement: Supplementary file 5 — Figure S3. Projection of reaction sets corresponding to SGS on the meta‐metabolic network. [file MBO3-5-106-s005.pdf]

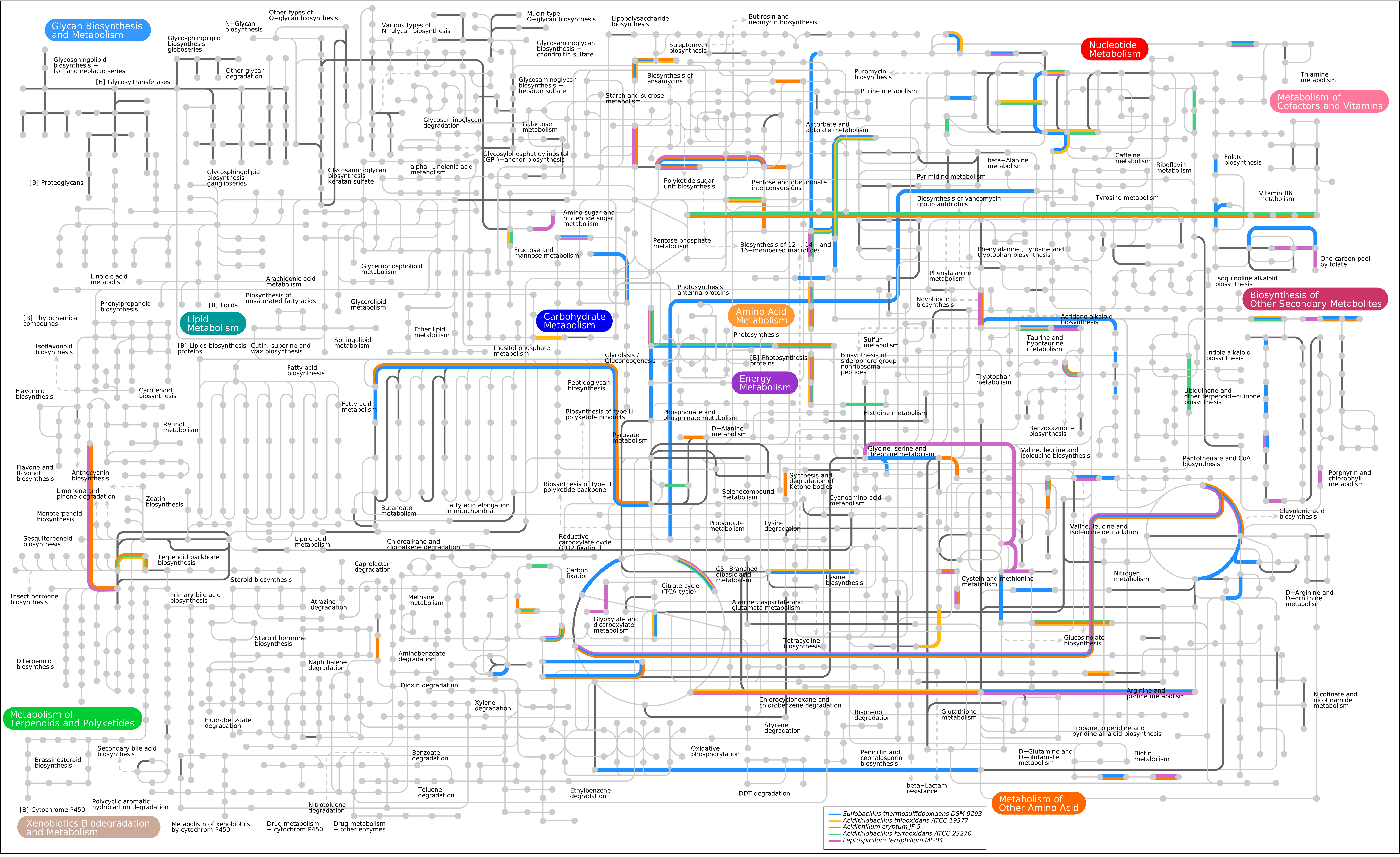

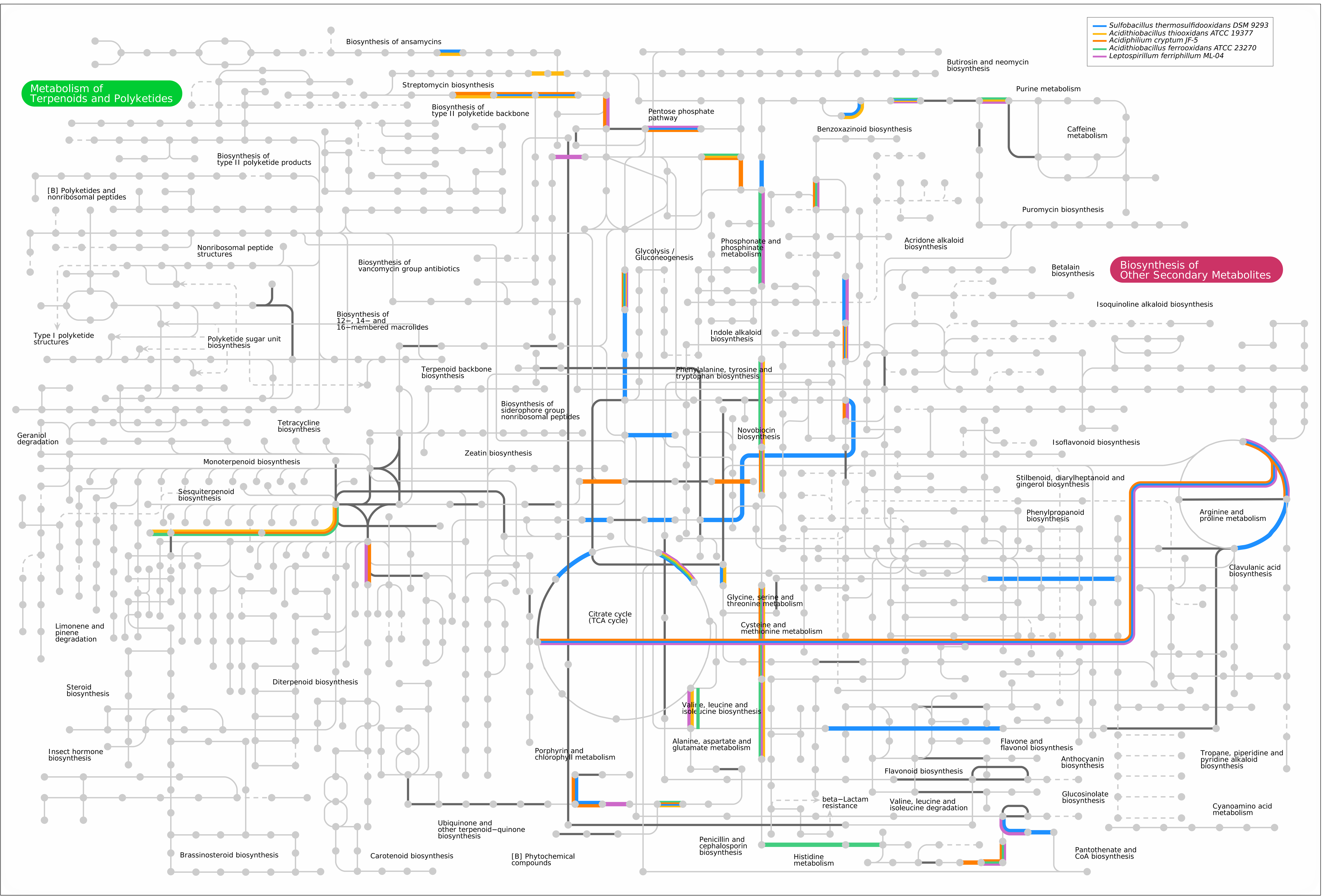

Figure S3: Projection of reaction sets corresponding to SGS on the meta-metabolic network. The upper part of the figure is the KEGG metabolic pathways map, and the lower part is the KEGG biosynthesis of secondary metabolites map. Notice that due to the conversion from Metacyc to KEGG some reactions do not appear in this figure. The blue, purple, green, orange and yellow edges refer respectively to *Sb. thermosulfidooxidans*, *L. ferriphilum*, *At. ferroxidans*, *A. cryptum* and *At. thiooxidans* reaction sets associated to their respective SGS. Dark grey edges illustrate reactions involved into the meta-metabolism of the five biomining bacteria whereas the light grey ones are reactions from the KEGG map not involved into our ecological system. Notice that, due to some binding incompatibilities between the Metacyc and KEGG databases, some reaction illustrations are and let appear misleading gaps larger than two reactions.
