# Supplementary material for: Putative bacterial interactions from metagenomic knowledge with an integrative systems ecology approach
Source: Microbiologyopen. 2015 Dec 17;5(1):106–17. doi: 10.1002/mbo3.315 (PMC4767419; doi:10.1002/mbo3.315)
Supplement: Supplementary file 6 — Figure S4. Superpathway of sulfate assimilation and cysteine biosynthesis from Metacyc (SULFATE‐CYS‐PWY). [file MBO3-5-106-s006.pdf]

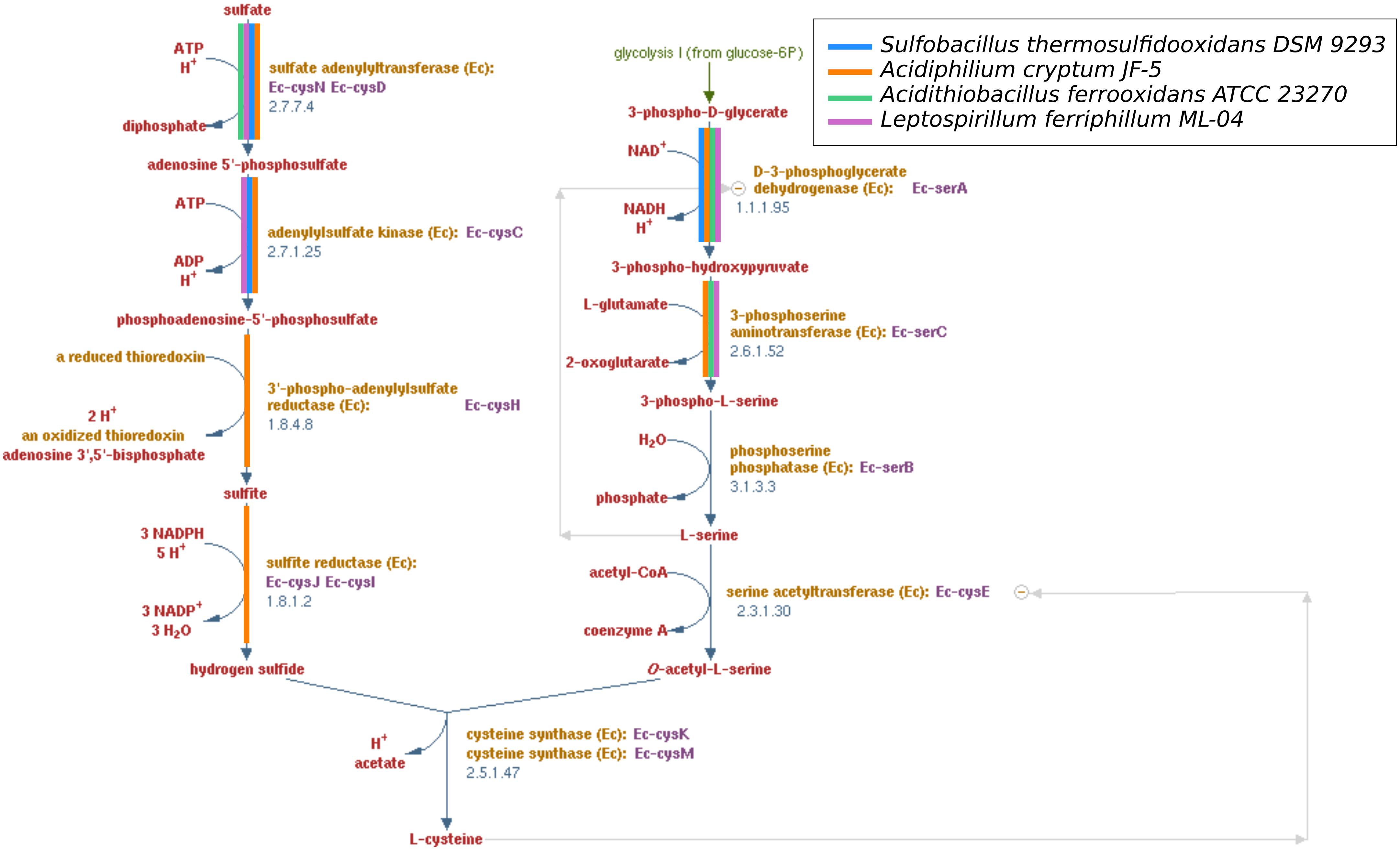

Figure S4: Superpathway of sulfate assimilation and cysteine biosynthesis from Metacyc (SULFATE-CYS-PWY). Each color band is the representation of a SGS. The orange ones are for *A. cryptum*, the purple one for *L. ferriphilum*, the blue ones for *Sb. thermosulfidooxidans* and the green one for *At. ferrooxidans*.
