# Supplementary material for: Putative bacterial interactions from metagenomic knowledge with an integrative systems ecology approach
Source: Microbiologyopen. 2015 Dec 17;5(1):106–17. doi: 10.1002/mbo3.315 (PMC4767419; doi:10.1002/mbo3.315)
Supplement: Supplementary file 7 — Figure S5. Pathway of NAD biosynthesis I (from aspartate) from Metacyc (PYRIDNUCSYN‐PWY). [file MBO3-5-106-s007.pdf]

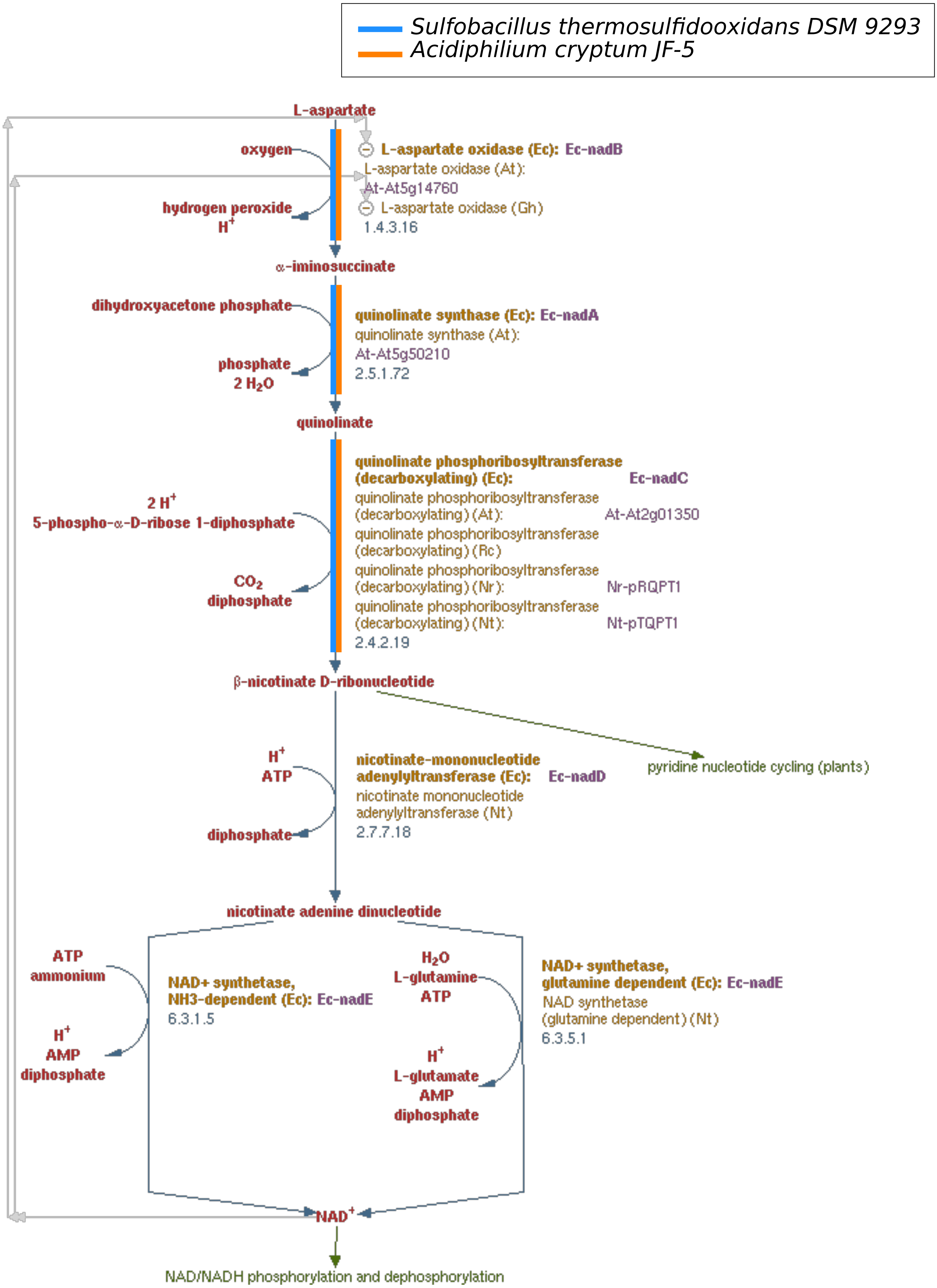

Figure S5: Pathway of NAD biosynthesis I (from aspartate) from Metacyc (PYRIDNUCSYN-PWY). Each color band is the representation of a SGS. The orange one is for *A. cryptum* and the blue one is for *Sb. thermosulfidooxidans*.
