# Supplementary material for: Putative bacterial interactions from metagenomic knowledge with an integrative systems ecology approach
Source: Microbiologyopen. 2015 Dec 17;5(1):106–17. doi: 10.1002/mbo3.315 (PMC4767419; doi:10.1002/mbo3.315)
Supplement: Supplementary file 8 — Figure S6. Pathway of NAD biosynthesis II (from tryptophan) from Metacyc (NADSYN‐PWY). [file MBO3-5-106-s008.pdf]

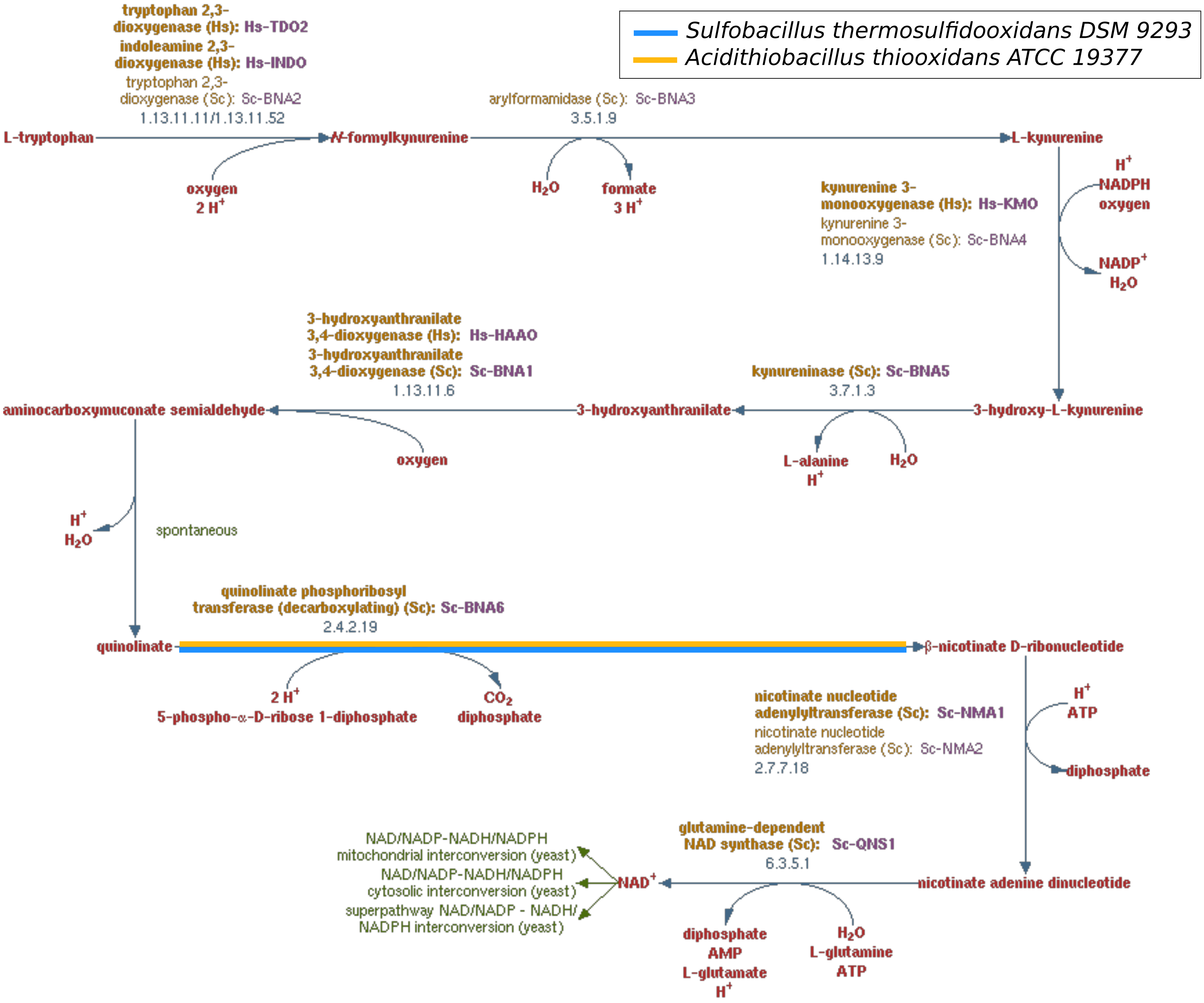

Figure S6: Pathway of NAD biosynthesis II (from tryptophan) from Metacyc (NADSYN-PWY). Each color band is the representation of a SGS. The yellow one is for *At. thiooxidans* and the blue one is for *Sb. Thermosulfidooxidans*.
