# Supplementary material for: Putative bacterial interactions from metagenomic knowledge with an integrative systems ecology approach
Source: Microbiologyopen. 2015 Dec 17;5(1):106–17. doi: 10.1002/mbo3.315 (PMC4767419; doi:10.1002/mbo3.315)
Supplement: Supplementary file 9 — Figure S7. Superpathway of heme biosynthesis from uroporphyrinogen‐III from Metacyc (PWY0‐1415). [file MBO3-5-106-s009.pdf]

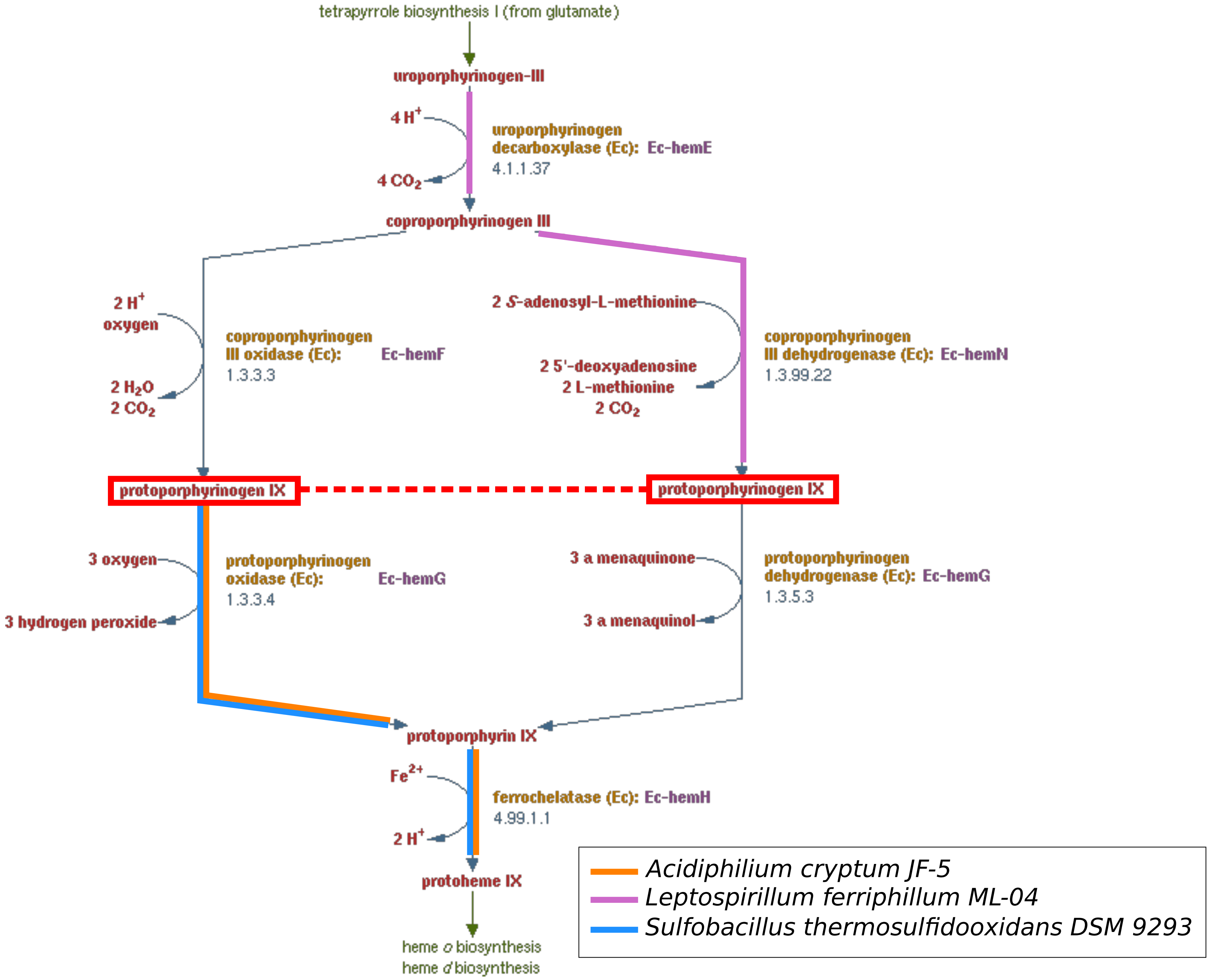

Figure S7: Superpathway of heme biosynthesis from uroporphyrinogen-III from Metacyc (PWY0-1415). Each color band is the representation of a SGS. The orange one is for *A. cryptum* the purple one for *L. ferriphilum*, and the blue one for *Sb. thermosulfidooxidans*. The red boxes linked with the red dash line highlight the fact that the SGS share a compound and are successive.
