# Supplementary material for: Putative bacterial interactions from metagenomic knowledge with an integrative systems ecology approach
Source: Microbiologyopen. 2015 Dec 17;5(1):106–17. doi: 10.1002/mbo3.315 (PMC4767419; doi:10.1002/mbo3.315)
Supplement: Supplementary file 11 — Figure S9. Pathway of heme biosynthesis from uroporphyrinogen‐III II from Metacyc (HEMESYN2‐PWY). [file MBO3-5-106-s011.pdf]

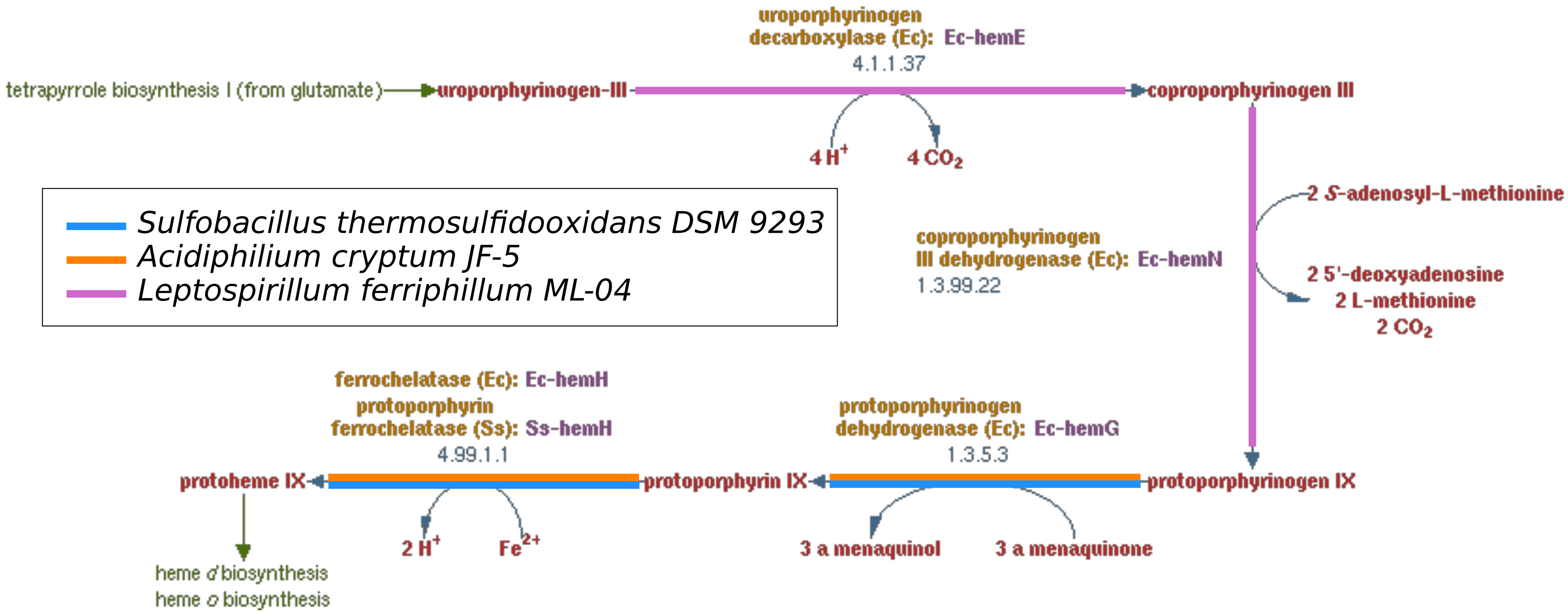

Figure S9: Pathway of heme biosynthesis from uroporphyrinogen-III II from Metacyc (HEMESYN2-PWY). Each color band is the representation of a SGS. The orange one is for *A. cryptum*, the purple one for *L. ferriphilum* and the blue one for *Sb. thermosulfidooxidans*. The whole pathway is covered by these SGS.
