# Supplementary material for: Putative bacterial interactions from metagenomic knowledge with an integrative systems ecology approach
Source: Microbiologyopen. 2015 Dec 17;5(1):106–17. doi: 10.1002/mbo3.315 (PMC4767419; doi:10.1002/mbo3.315)
Supplement: Supplementary file 12 — Figure S10. Superpathway of heme biosynthesis from glutamate from Metacyc (PWY‐5918). [file MBO3-5-106-s012.pdf]

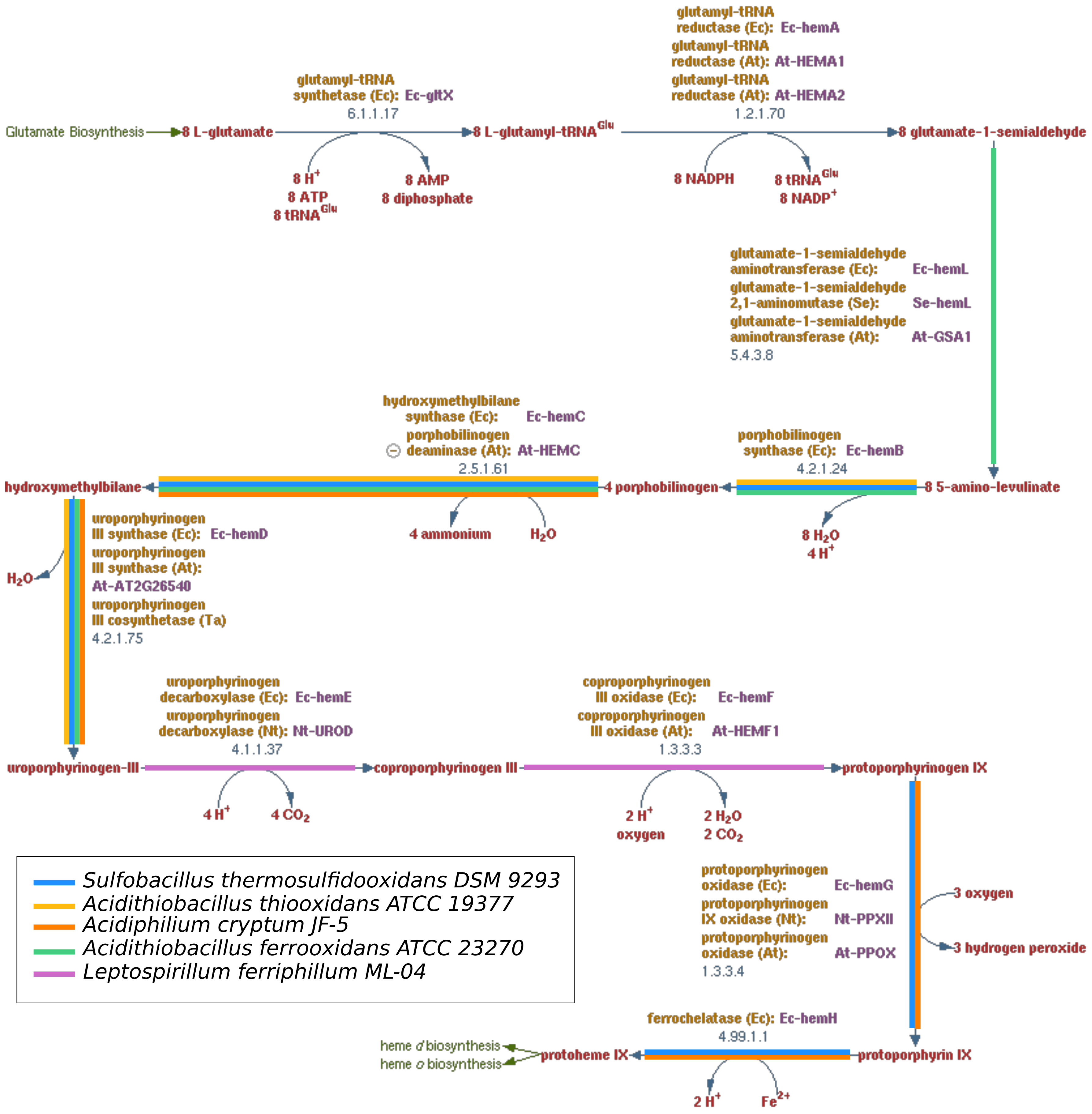

Figure S10: Superpathway of heme biosynthesis from glutamate from Metacyc (PWY-5918). Each color band is the representation of a SGS. The orange one is for *A. cryptum*, the purple one for *L. ferriphilum*, the blue one for *Sb. thermosulfidooxidans*, the yellow one for *At. thiooxidans* and the green one for *At. ferrooxidans*.
