# Supplementary material for: Putative bacterial interactions from metagenomic knowledge with an integrative systems ecology approach
Source: Microbiologyopen. 2015 Dec 17;5(1):106–17. doi: 10.1002/mbo3.315 (PMC4767419; doi:10.1002/mbo3.315)
Supplement: Supplementary file 13 — Figure S11. Pathway of glutathione biosynthesis from Metacyc (GLUTATHIONESYN‐PWY). [file MBO3-5-106-s013.pdf]

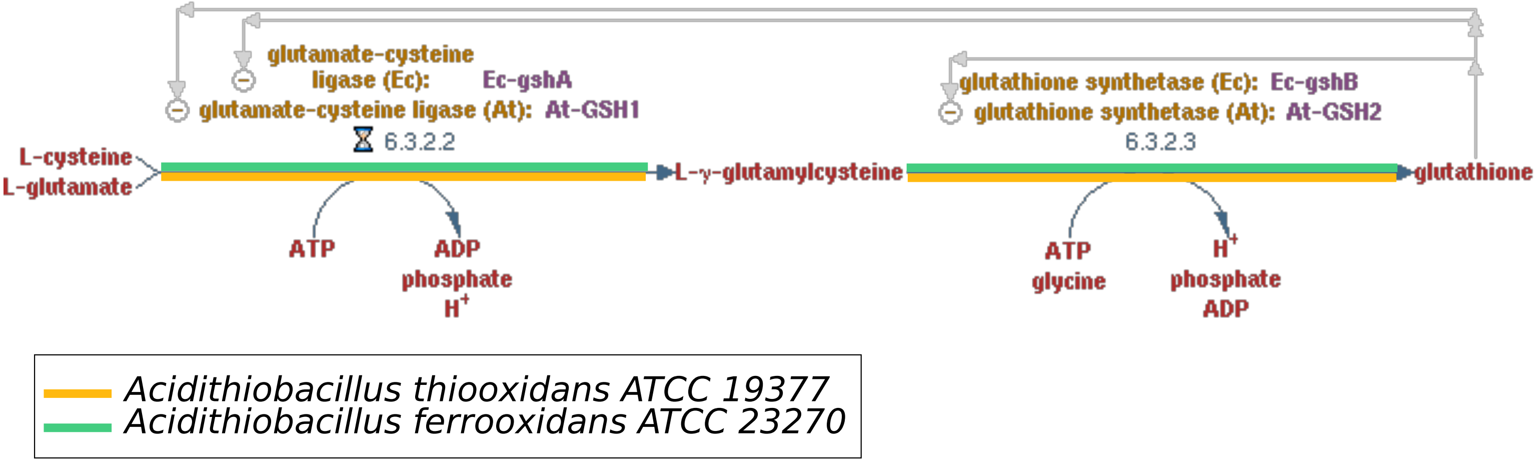

Figure S11: Pathway of glutathione biosynthesis from Metacyc (GLUTATHIONESYN-PWY). Each color band is the representation of some SGS. The yellow one is for *At. thiooxidans* and the green one for *At. ferrooxidans*.
